# Synthetic Naphthofuranquinone Derivatives Are Effective in Eliminating Drug-Resistant *Candida albicans* in Hyphal, Biofilm, and Intracellular Forms: An Application for Skin-Infection Treatment

**DOI:** 10.3389/fmicb.2020.02053

**Published:** 2020-08-26

**Authors:** Jia-You Fang, Kai-Wei Tang, Sien-Hung Yang, Ahmed Alalaiwe, Yu-Ching Yang, Chih-Hua Tseng, Shih-Chun Yang

**Affiliations:** ^1^Pharmaceutics Laboratory, Graduate Institute of Natural Products, Chang Gung University, Taoyuan City, Taiwan; ^2^Research Center for Food and Cosmetic Safety, Research Center for Chinese Herbal Medicine, Chang Gung University of Science and Technology, Taoyuan City, Taiwan; ^3^Department of Anesthesiology, Chang Gung Memorial Hospital, Taoyuan City, Taiwan; ^4^School of Pharmacy, College of Pharmacy, Kaohsiung Medical University, Kaohsiung, Taiwan; ^5^School of Traditional Chinese Medicine, Chang Gung University, Taoyuan City, Taiwan; ^6^Department of Traditional Chinese Medicine, Chang Gung Memorial Hospital, Taoyuan City, Taiwan; ^7^Department of Pharmaceutics, College of Pharmacy, Prince Sattam Bin Abdulaziz University, Al Kharj, Saudi Arabia; ^8^Department of Fragrance and Cosmetic Science, College of Pharmacy, Kaohsiung Medical University, Kaohsiung, Taiwan; ^9^Drug Development and Value Creation Research Center, Kaohsiung Medical University, Kaohsiung, Taiwan; ^10^Department of Medical Research, Kaohsiung Medical University Hospital, Kaohsiung, Taiwan; ^11^Department of Pharmacy, Kaohsiung Municipal Ta-Tung Hospital, Kaohsiung, Taiwan; ^12^Department of Cosmetic Science, Providence University, Taichung, Taiwan

**Keywords:** *C. albicans*, skin, drug resistance, naphthofuranquinone, biofilm, hypha

## Abstract

*Candida albicans* is the most common cause of fungal infection. The emergence of drug resistance leads to the need for novel antifungal agents. We aimed to design naphthofuranquinone analogs to treat drug-resistant *C. albicans* for topical application on cutaneous candidiasis. The time-killing response, agar diffusion, and live/dead assay of the antifungal activity were estimated against 5-fluorocytosine (5-FC)- or fluconazole-resistant strains. A total of 14 naphthofuranquinones were compared for their antifungal potency. The lead compounds with hydroxyimino (TCH-1140) or *O*-acetyl oxime (TCH-1142) moieties were the most active agents identified, showing a minimum inhibitory concentration (MIC) of 1.5 and 1.2 μM, respectively. Both compounds were superior to 5-FC and fluconazole for killing planktonic fungi. Naphthofuranquinones efficiently diminished the microbes inside and outside the biofilm. TCH-1140 and TCH-1142 were delivered into *C. albicans*-infected keratinocytes to eradicate intracellular fungi. The compounds did not reduce the *C. albicans* burden inside the macrophages, but the naphthofuranquinones promoted the transition of fungi from the virulent hypha form to the yeast form. In the *in vivo* skin mycosis mouse model, topically applied 5-FC and TCH-1140 reduced the *C. albicans* load from 1.5 × 10^6^ to 5.4 × 10^5^ and 1.4 × 10^5^ CFU, respectively. The infected abscess diameter was significantly decreased by TCH-1140 (3-4 mm) as compared to the control (8 mm). The disintegrated skin-barrier function induced by the fungi was recovered to the baseline by the compound. The data support the potential of TCH-1140 as a topical agent for treating drug-resistant *C. albicans* infection without causing skin irritation.

## Introduction

Pathogenic fungi have caused a huge threat to global health. The epidemiological data reveal that superficial fungi infection affects 25% of the worldwide population (Ghannoum et al., 2013). *Candida albicans* is the most frequent cause of fungal infection. It is estimated as the third most common infection in United States hospitals (Romo et al., 2017). As an opportunistic pathogen, *C. albicans* can accumulate in oral, vaginal, gastric, and cutaneous surfaces. Candidiasis is one of the most widespread classes of superficial fungal infection (Kühbacher et al., 2017). Cutaneous mycosis is also caused by the other dermatophytes, including *Microsporum*, *Epidermophyton*, and *Trichophyton* (Martinez-Rossi et al., 2017). In recent years, the increasing use of antifungal drugs for cutaneous mycosis treatment has led to the development of drug-resistant *C. albicans* (Larsen et al., 2018). This situation advocates the urgent need for novel antifungal agents.

There are only four classes of USFDA-approved antifungal drugs to treat candidiasis, including the structures of flucytosines, azoles, echinocandins, and polyenes. Naphthofuranquinones are a quinine subclass possessing some bioactivities such as antimicrobial, anti-inflammatory, and antitumor potencies (Tsang et al., 2018). Previous studies (Gershon and Shanks, 1975; Nagata et al., 1998; Neto et al., 2014; Rejiniemon et al., 2014; Hassan et al., 2016; Xie et al., 2016) demonstrated that naphthofuranquinones showed the capability to eradicate *Candida* species. We prepared a series of naphthofuranquinone derivatives, which displayed anti-inflammatory and anticancer activities (Tseng et al., 2009; Chien et al., 2010; Tsai et al., 2014). Recently we found that some of the compounds with a naphthofuranquinone backbone were antibacterial agents against methicillin-resistant *Staphylococcus aureus* (MRSA) (Yang et al., 2017). There are far fewer antifungal drugs than antibacterial drugs available in clinics. Because the incidence of dermatophytoses remains so high, there is still a need for efficient topical antifungal therapy. To address the necessity of novel antifungals, we have screened naphthofuranquinones developed in our lab to treat the *C. albicans* strains ATCC90029 and ATCC10231. ATCC90029 is resistant to 5-fluorocytosine (5-FC) while ATCC10231 is resistant to most of the antifungals, including fluconazole. ATCC90029 also can be regarded as the susceptible strain since it is only resistant to 5-FC but sensitive to most of the antifungal drugs. The emergence of resistance to antifungal drugs is usually due to the formation of biofilm, host cell residence, and filamentation, which increase the virulence of *C. albicans* (Monika et al., 2017). The ability of the naphthofuranquinone analogs to kill these difficult-to-treat *C. albicans* was also evaluated in this study.

The intracellular fungi killing was examined by employing keratinocytes and macrophages as the host cells infected by *C. albicans*. To explore the fungicidal mechanism of the compounds, morphological observation, total DNA, RNA, protein, and hypha-related genes were assessed to determine the mode of action on *C. albicans*. Finally, we compared the antifungal activity between the compound and 5-FC in the *in vivo* skin mycosis mouse model. Here, we demonstrated that topical delivery of the angular naphthofuranquinone with the hydroxyimino group (TCH-1140) remarkably mitigated abscess and the associated *C. albicans* burden.

## Materials and Methods

### Synthesis of Naphthofuranquinones

We synthesized 14 naphthofuranquinone-related compounds as shown inSupplementary Table S1. The protocols for synthesizing TCH-1139 and TCH-1199 were described in our previous investigation (Tseng et al., 2010). The synthetic protocols for the other compounds were shown in another study (Yang et al., 2019). The structures of all compounds were confirmed by ^1^H NMR, ^13^C NMR, and electrospray ionization mass spectrometry as exhibited previously.

### Fungal Strains

The strains of *C. albicans* employed in this work were ATCC90029 and ATCC10231 obtained from the Food Industry Research and Development Institute (Hsinchu, Taiwan). Both species were drug-resistant isolates.

### Minimum Inhibitory Concentration (MIC) and Minimum Fungicidal Concentration (MFC)

The antifungal activity of naphthofuranquinones was first estimated by MIC and MFC. A two-fold broth-dilution method was used to determine MIC as described previously (Chou et al., 2019). The treatment duration of the compounds to *C. albicans* was 16 h. MFC was detected as the lowest compound concentration for killing ≥99.9% of the fungi. The detailed procedures were represented in the previous study (Chou et al., 2019).

### Time-Response Fungus Eradication

The eradication of *C. albicans* by naphthofuranquinones during a 48-h period was evaluated in 96-well plates. The compounds at 2.9-11.6 μM were inoculated with test microbes (OD_600_ = 0.01) and incubated for 48 h at 37°C. The absorbance of each well was detected at 600 nm to determine the growth of *C. albicans* in a real-time mode.

### Inhibition Zone in Agar Diffusion Assay

This assay was performed by plating *C. albicans* (OD_600_ = 0.7) on the agar plate. Naphthofuranquinones at 0.7-2.9 μM (10 μl) were loaded onto the plate. After incubating for 12 h at 37°C, the clear zone diameter without fungi was calculated.

### Live/Dead Fungi Detection

The viability and death of *C. albicans* by treatment of naphthofuranquinones were monitored by Live/Dead BacLight^®^ kit. *C. albicans* was grown to OD_600_ = 0.1, and then treated with the compounds at 23.5-93.8 μM for 4 h. The strains stained by SYTO9 and propidium iodide (PI) were analyzed by fluorescence microscopy and flow cytometry. The detailed procedures were described earlier (Chou et al., 2019).

### Fungal Survival in Biofilm

The biofilm was established in a Cellview^®^ dish by incubating the microbes (OD_600_ = 0.1) in 1% glucose at 37°C for 24 h. The compounds at 11.6 μM were then incorporated into the biofilm for 24 h. The recovered *C. albicans* inside and outside the biofilm was loaded in an agar plate for 24 h to calculate CFU.

### Cytotoxicity of Keratinocytes and Macrophages

The vulture method for keratinocytes (HaCaT) was described in detail in our previous study (Lin et al., 2018). THP-1 cells were differentiated into macrophages by stimulation with 100 ng/ml phorbol myristate acetate for 36 h, followed by overnight incubation in fresh medium. Both 3-(4,5-dimethylthiazol-2-yl)-2,5-diphenyltetrazodium bromide (MTT) and CCK-8 assays were utilized to recognize cytotoxicity. The number of HaCaT and macrophages used in this experiment was 2 × 10^4^ and 1 × 10^5^ cells/well, respectively. The MTT assay was reported previously (Lin et al., 2018). The experimental procedures of CCK-8 were carried out based on the manufacturer’s protocol (BioTools, Taipei, Taiwan).

### Apoptosis

Analysis of Annexin V-fluorescein isothiocyanate (FITC) and PI cell staining was performed after 24 h of incubation of HaCaT or *C. albicans* in DMEM at 37°C and 5% CO_2_. The naphthofuranquinones dosed at 93.8 and 22.5 μM was used to treat HaCaT and *C. albicans*, respectively. After incubation, the cells were detached, washed with cold PBS, and treated with Annexin V-FITC and PI as suggested by the manufacturer (R&D Systems). The cells incubated with DMEM were employed as the control.

### Intracellular Fungus Eradication

Keratinocytes and macrophages differentiated from THP-1 were used as the host cells to estimate killing by naphthofuranquinones (11.7 μM). The fungal survival inside the host cells was appraised by colony-forming unit (CFU) counting and observed by confocal microscopy. DAPI and anti-*C. albicans* antibody/Alexa Fluor^®^ 488 goat anti-mouse IgG was utilized to stain the host cell nucleus and the fungi, respectively. The detailed processes were shown in the previous work (Alalaiwe et al., 2018).

### Morphological Visualization

*Candida albicans* morphology after treatment by naphthofuranquinones was monitored by scanning electron microscopy (SEM). The microbes at OD_600_ = 0.1 were treated with compounds at 11.7 μM for 24 h. The detailed processes of morphological observation were described earlier (Yang et al., 2016).

### Total Amounts of DNA, RNA, and Protein in Fungi

*Candida albicans* was grown to OD_600_ = 0.3. The compounds at 11.7 μM were incorporated with *C. albicans* suspension at 37°C for 4 h. The quantification of the total DNA, RNA, and protein in the fungi was performed using a Tools Bacterial and Fungal DNA Extraction kit, EasyPrep Total RNA kit (BioTools, Taipei, Taiwan), and Bio-Rad protein assay kit, respectively.

### Determination of Hypha-Related Genes

We employed real-time reverse transcription-polymerase chain reaction (RT-PCR) to analyze *efg1*, *ume6*, and *hgc1* in *C. albicans* after treatment of the compounds (11.7 μM). The RNA extraction and cDNA synthesis were conducted as described before (Yang et al., 2016). The cDNA was the template for RT-PCR amplification using gene-specific primers.

### Animals

Eight-week-old male Balb/c mice were used in the *in vivo* experiments. All protocols were performed in strict accordance with the recommendations set forth in the Guidelines for the Institutional Animal Care and Use Committee of Chang Gung University.

### *In vivo* Antifungal Activity of Naphthofuranquinones

All mice were prepared by shaving the dorsal fur. The mice were intradermally injected with 100 μl of the 5 × 10^6^ CFU ATCC10231 strain. The topically applied lead compound or 5-FC (2.9 mM) with a volume of 0.1 ml was administered onto the infection region every 24 h for 7 days. The skin-surface appearance was visualized by phenotypic and microscopic images after 7 days. The infected area was excised for homogenization. Fungus CFU in the infected site was estimated. TEWL was evaluated by Tewameter from 0 to 7 days post-injection.

### Histopathology

The mouse skin specimen was immersed in 10% formaldehyde and embedded in paraffin. The specimen was cut into a 5-μm thickness for hematoxylin and eosin (H&E) staining. We also examined immunoglobulin (Ig)G, interferon (IFN)-γ, interleukin (IL)-17, and Ly6G in the skin by immunohistochemistry (IHC). The skin sections were incubated with the related antibodies for 1 h, then incubated with biotinylated donkey anti-goat IgG for 20 min. The slices were visualized by optical microscopy.

### *In vivo* Skin Irritation

The vehicle with naphthofuranquinones or 5-FC (2.9 mM) was topically applied on the back of the mice for 5 days. The vehicle was replaced by a new one each day. The skin was examined for its microscopic appearance, transepidermal water loss (TEWL), erythema (a^∗^), skin-surface pH, and H&E-stained histology.

### Statistical Analysis

The data shown in this study presented the mean and standard deviation (S.D.). The significant difference between the various groups was checked by the Kruskal–Wallis test. The *post hoc* test for examining the individual difference was Dunn’s test. The significance was demonstrated as ^∗^ for *p* < 0.05, ^∗∗^ for *p* < 0.01, and ^∗∗∗^ for *p* < 0.001.

## Results

### Screening of Antifungal Activity of Naphthofuranquinones

**FIGURE 1:**
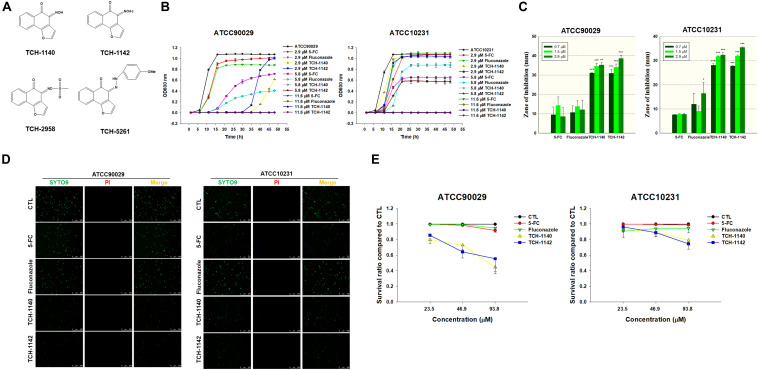
Determination of the antifungal activity of naphthofuranquinone derivatives against planktonic drug-resistant *C. albicans*: **(A)** the chemical structures of naphthofuranquinones; **(B)** time-killing curves of 5-FC, fluconazole, and naphthofuranquinones; **(C)** zone of inhibition measured from agar diffusion assay; **(D)** the planktonic live/dead *C. albicans* strains treated by the agents at 23.5 μM viewed under fluorescence microscopy; and **(E)** the survival rate of *C. albicans* measured by flow cytometry according to Supplementary Figure S2. All data are presented as the mean of three experiments ±SD. ***p < 0.001, **p < 0.01, *p < 0.05.

We designed naphthofuranquinones conjugated with imine moiety with angular or linear structures to test the antifungal effect. As a first step to screen the activity, we sought to identify the MFC against ATCC90029 (Table 1). Four analogs (TCH-1140, TCH-1142, TCH-2958, and TCH-5261) showed the MFC of <300 μM against 5-FC-resistant microbes. The structures of the four compounds are illustrated in Figure 1A. A negligible fungus inhibition was observed for the other analogs (MFC > 1,000 μM). The four derivatives were selected to further compare the MIC and MFC against ATCC90029 and ATCC10231 with 5-FC and fluconazole (Table 1). ATCC90029 was found to be susceptible to fluconazole but resistant to 5-FC. A contrary result was shown for ATCC10231. The MFC for fluconazole was 510-fold higher than the MIC against ATCC90029, indicating a fungistatic activity. ATCC90029 was most sensitive to TCH-1142, followed by TCH-1140. The MFC of both compounds against ATCC90029 was much less than against the positive controls. TCH-2958 and TCH-5261 were less effective than TCH-1140 and TCH-1142. TCH-1140 and TCH-1142 exhibited MFC of 2.9 and 2.4 μM against ATCC10231, respectively. Both compounds were found to be about 110- and 1,500-fold stronger than 5-FC and fluconazole, respectively.

**TABLE 1:** The MIC and MFC of naphthofuranquinones and the positive control against drug-resistant *C. albicans.*

	ATCC90029		ATCC10231	
		
	MIC (μM)	MFC (μM) 9683.2	MIC (μM) 37.8	MFC (μM)
5-Fluorocytosine	4841.6	9683.2	37.8	302.6
Fluconazole	4.0	2040.7	4081.4	4081.4
TCH-1140	1.5	2.9	2.9	2.9
TCH-1142	1.2	2.4	2.4	2.4
TCH-2958	134.1	268.2	268.2	268.2
TCH-5261	122.7	245.4	122.7	122.7

*MIC, minimum inhibitory concentration; MFC, minimum fungicidal concentration. Each value represents the 3 replicates.*

### Naphthofuranquinones Eradicate Planktonic *C. albicans*

TCH-1140 and TCH-1142 were the most promising naphthofuranquinones against drug-resistant *C. albicans*. The time-killing study offers information regarding the extent and rate of antifungal activity (Figure 1B). In the 5-FC-resistant microbes, 5-FC showed less fungal growth inhibition than the other agents tested. 5-FC demonstrated a sigmoidal response curve against planktonic ATCC90029. The positive controls inhibited ATCC90029 development in a concentration-dependent manner. The killing curve displayed that TCH-1140 and TCH-1142 completely inhibited ATCC90029 growth over a prolonged period (48 h). We observed a greater inhibition by TCH-1140 than TCH-1142 at 2.9 μM, the dose near the MFC of both compounds. However, a significant growth of *C. albicans* was observed after a 30-h treatment of both compounds at 2.9 μM. The time-killing curve showed that ATCC10231 was more resistant to the antifungal agents tested here than ATCC90029. ATCC10231 growth suppression was comparable between TCH-1140 and TCH-1142. The antifungal potency was estimated based on an agar diffusion assay (Figure 1C). 5-FC and fluconazole had a similar effect on the inhibition zone of both *C. albicans* strains. The zone diameter treated by different concentrations of the positive controls was comparable. There was a threefold increase of the inhibition diameter with naphthofuranquinone treatment compared with the positive controls. The inhibition diameter increased following the increase of the naphthofuranquinone dose, although this difference was not large.

We used live/dead staining with SYTO9 and PI to visualize the antifungal activity (Figure 1D). SYTO9 (green) stains the live microbes, whereas PI (red) penetrates the dead cells with a damaged membrane. The control group showed a diffuse distribution of live *C. albicans*. In the case of ATCC90029, SYTO9 was not decreased by 5-FC and fluconazole at 23.5 μM compared to the control. Naphthofuranquinone intervention led to a reduction of live ATCC90029 (green signal). On the other hand, 5-FC at 23.5 μM significantly decreased the fluconazole-resistant strain (ATCC10231), whereas fluconazole could not. A further decrease of the live ATCC10231 burden (green signal) was observed for naphthofuranquinones than 5-FC. The live/dead images of *C. albicans* treated with higher doses (46.9 and 93.8 μM) showed some SYTO9 reduction by the positive controls (Supplementary Figure S1). The green signal was nearly absent in the naphthofuranquinone-treated groups with higher concentrations. Though the live fungi were significantly restrained by the lead compounds, the PI signal was scanty. This suggests that the compounds eradicated fungi with limited membrane damage. A more precise reflection of live-cell detection is provided by flow-cytometry-based counting (Figure 1E). No fungus eradication was found in the control group (non-treatment). 5-FC and fluconazole (23.5-93.8 μM) diminished < 10% of *C. albicans* of both ATCC90029 and ATCC10231. On the other hand, naphthofuranquinones revealed a greater reduction of fungus viability compared to the positive controls. The lead compounds demonstrated a dose-dependent biocidal activity. TCH-1140 and TCH-1142 were equipotent. The lead compounds at 93.8 μM reduced the ATCC90029 and ATCC10231 viability by about 50 and 25%, respectively. The representative profiles of flow cytometry are depicted in Supplementary Figure S2.

### Naphthofuranquinones Eradicate Biofilm and Intracellular *C. albicans*

**FIGURE 2:**
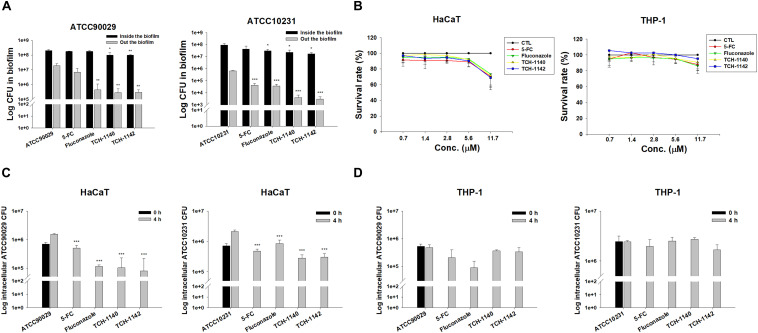
Determination of antifungal activity of naphthofuranquinone derivatives against biofilm and intracellular drug-resistant *C. albicans*: **(A)** *C. albicans* CFU outside and inside the biofilm; **(B)** the survival rate of keratinocytes and macrophages treated by 5-FC, fluconazole, and naphthofuranquinones measured by MTT assay; **(C)** the intracellular *C. albicans* CFU inside keratinocytes after treatment of 5-FC, fluconazole, and naphthofuranquinones; and **(D)** the intracellular *C. albicans* CFU inside macrophages after treatment of 5-FC, fluconazole, and naphthofuranquinones. All data are presented as the mean of three experiments ±SD (biological triplicate). ***p < 0.001, **p < 0.01.

Biofilm and intracellular residence are virulence mechanisms assisting *C. albicans* to resist antifungal drugs. We quantified the *C. albicans* amount inside and outside the biofilm after antifungal treatment (Figure 2A). The counting of the colony-forming unit (CFU) was log-transformed. 5-FC did not inhibit ATCC90029 viability inside and outside the biofilm. Fluconazole diminished CFU outside the biofilm by a 1.5 log reduction, but no inhibition was detected inside the biofilm. Both CFU inside and outside the ATCC90029 biofilm was significantly reduced by both lead compounds at a comparable level. In the case of ATCC10231, fluconazole diminished the number of live fungi inside the biofilm by about 12%. Both naphthofuranquinones inhibited fungus burden inside biofilm by about 50%. The positive controls and naphthofuranquinones lessened ATCC10231 CFU outside the biofilm by about 1 and 2 logs, respectively.

Keratinocytes are the main cells participating in the cutaneous immune response. Macrophages are the immune cells playing the major reservoirs for intracellular *C. albicans*. We employed both cells to examine the effect of lead compounds on intracellular fungus killing. First, the dose-dependent cytotoxicity against HaCaT (keratinocytes) and THP-1 (macrophages) was assessed using MTT and cell counting kit-8 (CCK-8) assays. The control in this experiment was the cells treated with DMSO vehicle (100% survival). The positive control drugs and TCH-1140 did not exhibit cytotoxicity against both cells (viability > 80%) in the MTT assay (Figure 2B). The same result was found in the CCK-8 assay (Supplementary Figure S3). The viability was reduced to 60-80% by TCH-1142, indicating a mild cytotoxicity. To elucidate if the treatment of TCH-1140 and TCH-1142 was associated with HaCaT cell death through apoptosis, the cultured keratinocytes were analyzed by flow cytometry after staining with FITC-labeled Annexin V and PI. Annexin V exhibits a high affinity to phosphatidylserine after its externalization from inner to the outer plasma membrane of apoptotic cells, while PI is a membrane-impermeant dye staining DNA. No significant increase in Annexin V and PI binding on the HaCaT surface was visualized after 24 h of compound treatment compared to the control cells (Supplementary Figures S4A,B), indicating no keratinocyte apoptosis occurred by naphthofuranquinone treatment at a high dose (93.8 μM).

The staining of Annexin V and PI was also performed for *C. albicans*. The results of ATCC90029 and ATCC10231 treated with TCH1140 show that the dot plots to lower right (LR) area is increased by this compound (Supplementary Figures S4C,D), manifesting an early apoptosis. The dot plots were also increased in the upper left (UL) area by TCH1140 intervention, indicating the late apoptosis. A similar result was observed for TCH1142 (Supplementary Figures S4E,F). These results demonstrated that the lead compounds caused both early and late apoptosis for killing *C. albicans*. We then evaluated whether the compounds could kill intracellular *C. albicans* in HaCaT or THP-1. The infected cells were treated by the compounds for 4 h after unphagocytosed fungus removal. All agents tested significantly reduced ATCC90029 in keratinocytes (Figure 2C). Fluconazole and naphthofuranquinones exhibited a 1-log greater anti-ATCC90029 effect than 5-FC. The effect among fluconazole, TCH-1140, and TCH-1142 was comparable. In the case of ATCC10231, naphthofuranquinones demonstrated a greater capability to kill fungi than fluconazole. None of the agents revealed a significant *C. albicans* CFU reduction in the macrophages (Figure 2D).

**FIGURE 3:**
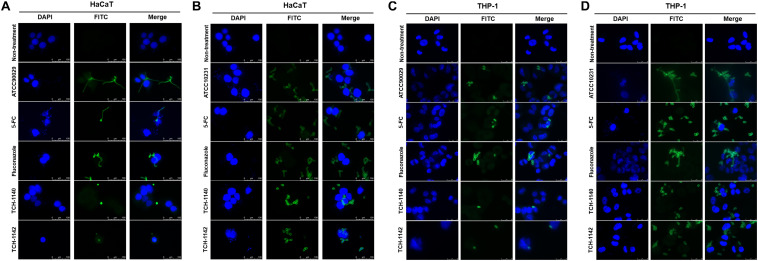
The confocal microscopic images of *C. albicans* (green) in the host cells (blue) after treatment of 5-FC, fluconazole, and naphthofuranquinones: **(A)** ATCC90029-infected keratinocytes; **(B)** ATCC10231-infected keratinocytes; **(C)** ATCC90029-infected macrophages; and **(D)** ATCC10231-infected macrophages.

Encouraged by these findings, we visualized the morphology of *C. albicans* in the host cells after treatment with lead compounds (Figure 3). The infected cells were exposed to fluorescently labeled *C. albicans* (green) and DAPI (blue) for monitoring by confocal microscopy. The images indicated that both fungal strains in HaCaT or THP-1 showed significant hyphae. Some fungi were present outside the host cells, suggesting a possible escape from the cells during a 4-h incubation. These filamentous hyphae in keratinocytes were unaffected by 5-FC and fluconazole (Figures 3A,B). On the other hand, the hypha filament could be shortened or transferred into pseudohypha form by 5-FC and fluconazole in macrophages (Figures 3C,D). The inhibited fungal filamentation was observed in naphthofuranquinone-treated keratinocytes. The filamentous fungi could change into unicellular yeast or pseudohypha form after lead compound intervention in macrophages. The large field view of ATCC10231 infected in HaCaT and THP-1 under confocal microscopy is shown in Supplementary Figures S5A,B, respectively.

### Elucidation of Possible Antifungal Mechanisms of Naphthofuranquinones

**FIGURE 4:**
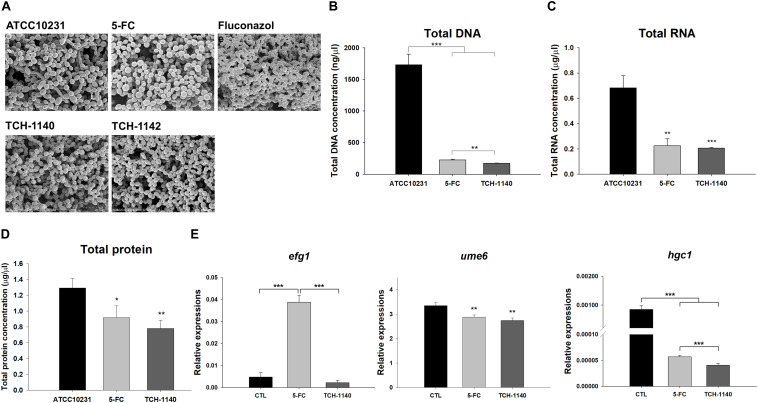
Antifungal mechanisms of naphthofuranquinones: **(A)** morphological changes of ATCC10231 viewed under TEM; **(B)** total DNA amount in ATCC10231; **(C)** total RNA amount in ATCC10231; **(D)** total protein amount in ATCC10231; and **(E)** the expression of hypha-related genes *efg1*, *ume6*, and *hgc1*. All data are presented as the mean of three experiments ±SD. ***p < 0.001, **p < 0.01, *p < 0.05.

Antifungal mechanisms of the lead compounds were investigated. We began this study by SEM (Figure 4A). ATCC10231 in dense biofilm form was observed to show both yeast and hypha morphologies. The hypha form was absent with treatment of the positive controls and naphthofuranquinones. The fungal shape and surface remained intact after 5-FC intervention. Fluconazole and naphthofuranquinones generated a morphological alteration on the fungal surface, demonstrating the membrane destabilization. The wrinkled and rough surfaces with cavities indicated a phenotype of dead microbes. The size of some *C. albicans* decreased when exposed to TCH-1140 and TCH-1142, which was indicative of fungal death. Next, we quantified the total DNA, RNA, and protein in 5-FC- and TCH-1140-treated ATCC10231 (Figures 4B–D). TCH-1142 was not used in the following experiments because of its higher toxicity toward mammalian cells than TCH-1140. The DNA load in *C. albicans* was reduced by 87 and 90% in the 5-FC and TCH-1140 groups, respectively. The RNA and protein analyses also indicated a depressed amount after treatment of 5-FC and TCH-1140. The reduction between 5-FC and hydroxyimino-conjugated naphthofuranquinone was comparable.

To explore the role of TCH-1140 on hypha suppression, the expression of hypha-related genes *efg1*, *ume6*, and *hgc1* at the transcriptional level was measured (Figure 4E). TCH-1140 reduced *efg1* expression as compared to the control although the statistical significance was not achieved. Surprisingly, 5-FC elevated *efg1* by eightfold. The gene *ume6* was found to be significantly downregulated by 5-FC and TCH-1140. The same result was observed in *hgc1*, with TCH-1140 showing greater inhibition.

### TCH-1140 Mitigates *C. albicans* Burden *In vivo*

**FIGURE 5:**
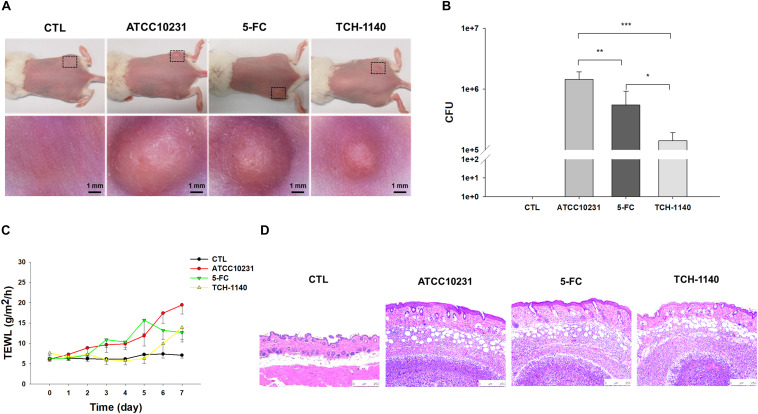
*In vivo* topical application of TCH-1140 against ATCC10231: **(A)** the skin surface of ATCC10231-infected mice after treatment of 5-FC or TCH-1140 viewed under phenotypic and microscopic images; **(B)** ATCC10231 CFU in mouse skin after treatment of 5-FC or TCH-1140; **(C)** TEWL of ATCC10231-infected mice after treatment of 5-FC or TCH-1140; and **(D)** histological observation of ATCC10231-infected mouse skin biopsy stained by H&E. All data are presented as the mean of six experiments ±SD. ***p < 0.001, **p < 0.01, *p < 0.05.

A mouse model of skin mycosis induced by ATCC10231 was used to rate the efficacy of TCH-1140 *in vivo*. After induction of cutaneous candidiasis, the nidus showed pustule, edema, and inflammation of the skin with an abscess diameter of about 8 mm (Figure 5A). 5-FC treatment restricted the abscess diameter to 5-6 mm. The severity of the lesion was further decreased in the TCH-1140-treated animals with the diameter of 3-4 mm, indicating a significant wound healing. The same tendency was found in the fungal count (Figure 5B). TCH-1140 achieved a 1-log CFU reduction as compared to the infection group without treatment. TEWL was detected daily to evaluate skin-barrier function (Figure 5C). *C. albicans* infection produced cutaneous inflammation to disintegrate the barrier feature for increasing TEWL. 5-FC reduced this elevation from day 6. The baseline TEWL remained up to day 5 by TCH-1140; however, some barrier disruption was observed after day 6. A histopathological assay of the abscess of ATCC10231-infected skin showed a large *C. albicans* burden under the subcutis (Figure 5D). Some fungi invaded to the dermis and subcutis. Inflammatory cell infiltration was also visualized in the viable skin. The animals receiving 5-FC displayed an improvement in the fungal load and immune cell accumulation. A further mitigation of the fungal burden and infiltration was detected after TCH-1140 administration.

Some proinflammatory mediators expressed in the skin were observed by IHC. The IgG expression was higher in the infected abscess and dermis compared with the healthy skin (Supplementary Figure S6A). The IgG upregulation by infection could be improved by 5-FC and TCH-1140. The level of IFN-γ in the dermis and around the abscess after ATCC10231 injection was higher than in the control animal (Supplementary Figure S6B). No significant IFN-γ reduction was observed by 5-FC treatment. TCH-1140 significantly downregulated IFN-γ in cutaneous tissue compared to the infected animal, whereas the expression in the margin of abscess seemed to increase. IL-17 was extensively distributed in the abscess and subcutaneous region of ATCC10231-infected animals (Supplementary Figure S6C). This distribution was not diminished by antifungal agent application. The neutrophil infiltration in the skin was observed by Ly6G staining (Supplementary Figure S6D). Some focal neutrophils in the dermis were seen after *C. albicans* injection. This focal accumulation was still detected after 5-FC treatment. The focal Ly6G was minimal in the TCH-1140-treated dermis.

### TCH-1140 Elicits a Negligible Skin Irritation

**FIGURE 6:**
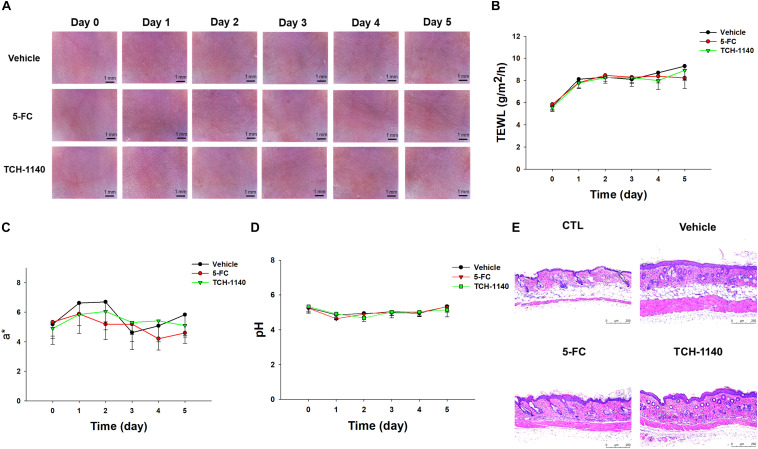
Skin tolerance examination of mouse skin by a 5-day treatment of topically applied TCH-1140: **(A)** the skin surface of mice viewed under handheld digital magnifier; **(B)** TEWL of mouse skin after treatment of 5-FC or TCH-1140; **(C)** erythema (a*) of mouse skin after treatment of 5-FC or TCH-1140; **(D)** pH of mouse skin after treatment of 5-FC or TCH-1140; and **(E)** histological observation of mouse skin biopsy stained by H&E. All data are presented as the mean of six experiments ±SD.

5-FC or TCH-1140 was topically applied on healthy mouse skin to check the possibility of inducing irritation. No visible redness, scaling, or edema was observed on the cutaneous surface treated with the compounds as compared to vehicle control for 5 days (Figure 6A). No disrupted barrier function (TEWL), erythema (a^∗^), or skin surface pH was changed after administration of the compounds (Figures 6B–D). TEWL was increased from 6 to 8 g/m^2^/h at day 1 for all groups. This could be due to the capability of the aqueous vehicle to hydrate the stratum corneum. The 5-FC and TCH-1140 groups exhibited cutaneous histology similar to the vehicle control (Figure 6E).

## Discussion

*Candida albicans* is a primary fungus responsible for skin and mucous infection. Drug-resistant fungus strains present an important clinical concern. As a result, novel agents are urgently needed to resolve the problem of resistance. In this study, thorough analyses were conducted to evaluate the capability of *C. albicans* eradication by naphthofuranquinones prepared in our lab. We found that TCH-1140 and TCH-1142 were effective in killing drug-resistant *C. albicans* either in planktonic form or in the highly virulent forms (hypha, biofilm, and host cell residence). The *in vivo* effect of the lead compound in the cutaneous candidiasis mouse model together with acceptable safety reinforce the potential as a candidate in the development of an antifungal agent.

After screening the antifungal activity of naphthofuranquinones by MFC, the angular-structured compounds substituted by hydroxyimino (TCH-1140) and *O*-acetyl oxime (TCH-1142) moieties at the 4-position showed the greatest potency. All others had negligible activity. The linear naphthofuranquinone structures totally lost the antifungal activity. The conjugation of the hydrazino group at 4-position also eliminated the activity. We selected TCH-1140 and TCH-1142 for further comparison with 5-FC and fluconazole. The MIC of TCH-1140 and TCH-1142 against ATCC90029 was 1.5 μM (0.32 μg/ml) and 1.2 μM (0.31 μg/ml), respectively. These values were greatly lower than the other naphthoquinone derivatives reported earlier such as 2-methyl-5-hydroxynaphtho[2,3-*b*]furan-4,9-dione (2-8 μg/ml), phenanthrenequinone (60 μg/ml), 2-[iodomethyl]-2,3-dihydronaphtho[2,3-*b*]furan-4,9-dione (12 μg/ml), and plumbagin (8 μg/ml) (Nagata et al., 1998; Neto et al., 2014; Rejiniemon et al., 2014; Hassan et al., 2016), although the *C. albicans* strains used were different among the different studies. This suggested the potent antifungal activity of the naphthofuranquinones developed in this work. Clinical use of 5-FC and fluconazole is limited due to toxicity concerns and resistance emergence. 5-FC is a nucleoside analog impairing DNA and protein synthesis. Its side effects are bone-marrow suppression, diarrhea, and skin rash (Meletiadis et al., 2008). Fluconazole is the agent most commonly used to treat *Candida* infection. The overuse of fluconazole has developed a significant incidence of resistance, resulting in therapeutic failure (Reis de Sá et al., 2017). It is appreciated that *C. albicans* is considered resistant at the MIC ≥ 210 μM (Ghannoum and Rice, 1999). We recognized that ATCC10231 was resistant to fluconazole, while ATCC90029 was sensitive to this drug. A contrary tendency was found for 5-FC. An agent is regarded as fungicidal and fungistatic when the MFC/MIC ratio is less or higher than 4, respectively (Ganan et al., 2019). Our results demonstrated that naphthofuranquinones were fungicidal. An ideal biocidal agent should kill the microbes quickly to avoid the opportunity for resistance development (Ng et al., 2017). Our time-killing curve showed a rapid fungicidal effect with the use of TCH-1140 and TCH-1142.

The naphthofuranquinones evoked fungal wall and/or membrane disturbance based on SEM, indicating the occurrence of compromised membrane integrity. However, this damage could be regarded as mild since PI could not permeate into the cytoplasm as observed in the live/dead assay. Naphthofuranquinone intervention interfered with the total DNA, RNA, and protein synthesis in the fungi. The lead compounds might penetrate the membrane into the fungal cytoplasm, producing killing by DNA replication inhibition but not the direct membrane disintegration. Drug efflux is an important resistance mechanism, especially for fluconazole resistance (Berkov and Lockhart, 2017). The superior eradication of the drug-resistant *C. albicans* by naphthofuranquinones compared to that by fluconazole could be due to the inhibition of the efflux pump regulated by some genes such as *mdr1*. The mechanisms of *C. albicans* death elicited by the lead compounds are still not completely understood. It should also be noticeable that the *C. albicans* growth was elevated after a 30-h treatment of lead compounds at 2.9 μM in the time-response test. The strains might gain resistance toward the compounds after a long-term intervention. Further study is needed to explore the details.

*Candida* species are difficult to treat as they invade host cells. This is a manifestation of drug resistance. More than 60% of the antimicrobials failed to manage intracellular microbes (Murali et al., 2018). Both keratinocytes and macrophages are the host cells that are invaded by topical fungi infecting the skin (Lopez et al., 2014). Although macrophages are the innate immune systems for eliminating *C. albicans*, most of the fungi survive inside the macrophages with only a portion of the population being killed (Diez-Orejas and Fernández-Arenas, 2008). Here, we demonstrated that TCH-1140 could enter the keratinocytes to kill *C. albicans* without affecting host-cell viability. This intracellular elimination by naphthofuranquinones was better than 5-FC and fluconazole. The lead compounds did not arrest *C. albicans* growth in the macrophages. Rather, they drove the fungal transition from the pathologic hypha to the pseudohypha or yeast. The naphthofuranquinones can be anti-virulence agents. Engulfment of yeast *C. albicans* by keratinocytes and macrophages induces the production of hypha, which exerts a virulent action (Kano et al., 2003; Arnaud et al., 2009). The hyphal formation allows the puncture of the macrophage membrane, leading to the escape of *C. albicans* from the cells (Wartenberg et al., 2014). This was the reason why we saw some microbes outside the infected macrophages. The naphthofuranquinones maintained the fungi in the yeast or pseudohypha type, which was beneficial to preserving the function of macrophages for eliminating fungi.

The development of resistance to antifungal drugs often occurs in filamentous fungi. *efg1*, *ume6*, and *hgc1* are transcription regulators associated with hypha formation. The transcription factor *efg1* functions to promote filamentous growth (Park et al., 2020). *ume6* is a downstream target of hypha-growth signaling (Zeidler et al., 2009). This key regulator functions downstream of *efg1* and upstream of *hgc1*. The experimental data demonstrated that TCH-1140 suppressed the transcription of these genes. The upregulation of *efg1* by 5-FC was surprising. *efg1* appears to be an upstream regulator in the network of filamentation. Besides the involvement of the hypha form, *efg1* also acts as the regulator of cell wall proteins, adhesion, aspartic protease, and antifungal resistance (Gow et al., 2012). 5-FC stimuli might cause *efg1* upregulation in response to the environmental change because of the complex role of this gene in adapting morphogenetic transition.

Both morphogenesis and biofilm are the important virulences of drug-resistant *C. albicans*. Hyphal elements are the main structures embedded in mature *C. albicans* biofilm (Vavala et al., 2013). The biofilm presents a permeation barrier to antifungal drugs and phagocytes (Roscetto et al., 2018). 5-FC and fluconazole are less sensitive to treat biofilm-associated invasion. The evidence shows that the drug concentration needed to eradicate biofilm fungi is 5-8 times greater than that needed to eradicate planktonic fungi (Mathé and Van Dijck, 2013). The antifungal agents with the ability to diminish biofilm microbes are promising for reducing colonization on the skin and in the mucus (Rejiniemon et al., 2014). We demonstrated that the synthetic naphthofuranquinones showed their activity against *C. albicans* inside the mature biofilm. Although a statistically significant CFU reduction was obtained, this killing by the compounds was still limited. The high cell density inside the biofilm has markedly lowered susceptibility to all drugs (Mathé and Van Dijck, 2013). The dispersed *C. albicans* from biofilm displays increased virulence, filamentation, and drug resistance, which are responsible for invasive infection (Wall et al., 2019). The naphthofuranquinones were fungicidal not only against *C. albicans* inside the biofilm but also against the microbes dispersed from the biofilm. The dispersal inhibition by TCH-1140 and TCH-1142 was superior to that in the positive controls.

The emergence of *C. albicans* strains resistant to current agents has contributed to an increase in the skin infection reported in humans. Our results manifested that TCH-1140 was superior to 5-FC for ameliorating drug-resistant *C. albicans*-induced infection and inflammation. TCH-1140 also recovered skin-barrier capacity disrupted by fungi. *C. albicans* infection is a result of a coordinated battle with host cells. The recognition of *C. albicans* by skin cells and innate immune cells induces the production of proinflammatory mediators. A previous study (Zhang et al., 2018) proved that *C. albicans* infection induced candidiasis with IL-17 and IFN-γ expression. Th17 cells are vital in the defense against fungal infection (Engelhardt and Grimbacher, 2012). IL-17 overexpression by Th17 cells can cause inflammation. IL-17 clears *C. albicans* through neutrophil recruitment for killing the pathogens by releasing antimicrobial peptides or phagocytosis (Kashem and Kaplan, 2016). The phagocytosis is an early line of defense for clearing fungi. The skin infected with ATCC10231 and topically treated with TCH-1140 showed a reduction of the fungal burden and focal neutrophil accumulation. We hypothesized that the inhibition of fungal colonization by TCH-1140 in the early infection stage attenuated the host immune response and inflammation. IgG reveals an important role in the immunity via raising fungal phagocytosis (Munawara et al., 2017). We also showed a significant reduction of IgG distribution in the skin and abscess regions infected by ATCC10231.

IFN-γ is chiefly secreted by keratinocytes to play a key role in the defense against *C. albicans* (Shiraki et al., 2008). TCH-1140 suppressed IFN-γ in the dermis, suggesting inflammation mitigation. However, IFN-γ expression increased around the fungus-induced abscess by TCH-1140. This could be because the increase of IgG by macrophages in the presence of *C. albicans* results in the decrease of IFN-γ, the Th1-related response (Munawara et al., 2017). Gow et al. (2012) also demonstrated that fungal hypha fails to evoke the Th1 immune response. The assessment of toxicity is principal in the development of new therapeutic drugs for clinical use. We were inspired by the skin irritation test to confirm that topical TCH-1140 was toxic toward drug-resistant fungi but tolerable to the skin.

## Conclusion

After screening a series of naphthofuranquinones, TCH-1140 and TCH-1142 were proven to be the most potent for eradicating drug-resistant *C. albicans*. Both compounds were active against yeast, filamentous, and biofilm *C. albicans* strains that were resistant to 5-FC or fluconazole. The lead compounds could deliver into the host cells, leading to a marked clearance of intracellular fungi. The resistant fungus infection and the associated inflammation in the skin were mitigated by TCH-1140. Our findings suggested that TCH-1140 could be a candidate for the treatment of skin mycosis. There is room for further structural modification in naphthofuranquinones for lead optimization of the potentiating activity. Naphthofuranquinone analogs do warrant further investigation as a topical agent for treating fungus-infected skin lesions.

## Data Availability Statement

The raw data supporting the conclusions of this article will be made available by the authors, without undue reservation, to any qualified researcher.

## Ethics Statement

The animal study was reviewed and approved by Institutional Animal Care and Use Committee of Chang Gung University.

## Author Contributions

J-YF initiated the study and drafted the manuscript. K-WT involved in the design of all experiments. S-HY, S-CY, and Y-CY carried out the experiments. AA analyzed data and wrote the manuscript. S-CY supervised the entire project. C-HT reviewed critically and approved the final manuscript. All authors read and approved the final manuscript.

## Conflict of Interest

The authors declare that the research was conducted in the absence of any commercial or financial relationships that could be construed as a potential conflict of interest.

## Supplementary Material

FIGURE S1 The planktonic live/dead *C. albicans* strains treated by the agents at 46.9 or 93.8 μM viewed under fluorescence microscopy: **(A)** ATCC90029 treated by 5-FC, fluconazole, and naphthofuranquinones at 46.9 μM. **(B)** ATCC10231 treated by 5-FC, fluconazole, and naphthofuranquinones at 46.9 μM. **(C)** ATCC90029 treated by 5-FC, fluconazole, and naphthofuranquinones at 93.8 μM. **(D)** ATCC10231 treated by 5-FC, fluconazole, and naphthofuranquinones at 93.8 μM.

FIGURE S2 The flow cytometry of *C. albicans* viability treated by 5-FC, fluconazole, and naphthofuranquinones : **(A)** ATCC90029; and **(B)** ATCC10231. FL1 (*x*-axis) represents SYTO9 staining. FL3 (*y*-axis) represents PI staining.

FIGURE S3 The survival rate of keratinocytes and macrophages treated by 5-FC, fluconazole, and naphthofuranquinones measured by CCK-8 assay: **(A)** keratinocytes; and **(B)** macrophages. All data are presented as the mean of three experiments ±S.D.

FIGURE S4 The flow cytometry of keratinocytes and *C. albicans* stained by Annexin V and PI to detect the early or late apoptosis: **(A)** keratinocytes treated with TCH-1140 at 93.8 μM; **(B)** keratinocytes treated with TCH-1142 at 93.8 μM; **(C)** ATCC90029 treated with TCH-1140 at 23.5 μM; **(D)** ATCC10231 treated with TCH-1140 at 23.5 μM; **(E)** ATCC90029 treated with TCH-1142 at 23.5 μM; and **(F)** ATCC10231 treated with TCH-1142 at 23.5 μM. All data are presented as the mean of three experiments ±S.D. LL, lower left; LR, lower right; UL, upper left; UR, upper right.

FIGURE S5 The large field view of confocal microscopic images of ATCC10231 after treatment of 5-FC, fluconazole, and naphthofuranquinones: **(A)** ATCC10231-infected keratinocytes; and **(B)** ATCC10231-infected macrophages.

FIGURE S6 The IHC staining of ATCC10231-infected mouse skin after treatment of 5-FC or TCH-1140: **(A)** IgG; **(B)** IFN-γ; **(C)** IL-17, and **(D)** Ly6G.

## References

[B1] Alalaiwe A. Wang P. W. Lu P. L. Chen Y. P. Fang J. Y. Yang S. C. (2018). Synergistic anti-MRSA activity of cationic nanostructured lipid carriers in combination with oxacillin for cutaneous application. *Front. Microbiol.* 9:1493. 10.3389/fmicb.2018.01493 30034381 PMC6043785

[B2] Arnaud M. B. Costanzo M. C. Shah P. Skrzypek M. S. Sherlock G. (2009). Gene ontology and the annotation of pathogen genomes: the case of *Candida albicans*. *Trends Microbiol.* 17 295–303. 10.1016/j.tim.2009.04.007 19577928 PMC3907193

[B3] Berkov E. L. Lockhart S. R. (2017). Fluconazole resistance in *Candida* species: a current perspective. *Infect. Drug Resist.* 10 237–245. 10.2147/idr.s118892 28814889 PMC5546770

[B4] Chien C. M. Lin K. L. Su J. C. Chuang P. W. Tseng C. H. Chen Y. L. (2010). Naphtho[1,2-*b*]furan-4,5-dione induces apoptosis of oral squamous cell carcinoma: involvement of EGF receptor/PI3K/Akt signaling pathway. *Eur. J. Pharmacol.* 636 52–58. 10.1016/j.ejphar.2010.03.030 20371243

[B5] Chou W. L. Lee T. H. Huang T. H. Wang P. W. Chen Y. P. Chen C. C. (2019). Coenzyme Q0 from *Antrodia cinnamomea* exhibits drug-resistant bacteria eradication and keratinocyte inflammation mitigation to ameliorate infected atopic dermatitis in mouse. *Front. Pharmacol.* 10:1445. 10.3389/fmicb.2018.01445 31849685 PMC6901829

[B6] Diez-Orejas R. Fernández-Arenas E. (2008). *Candida albicans*-macrophage interactions: genomic and proteomic insights. *Future Microbiol.* 3 661–681. 10.2217/17460913.3.6.661 19072183

[B7] Engelhardt K. R. Grimbacher B. (2012). Mendelian traits causing susceptibility to mucocutaneous fungal infections in human subjects. *J. Allergy Clin. Immunol.* 129 294–305. 10.1016/j.jaci.2011.12.966 22284928

[B8] Ganan M. Lorentzen S. B. Agger J. W. Heyward C. A. Bakke O. Knutsen S. H. (2019). Antifungal activity of well-defined chito-oligosaccharide preparations against medically relevant yeasts. *PLoS One* 14:e0210208. 10.1371/journal.pone.0210208 30620751 PMC6324834

[B9] Gershon H. Shanks L. (1975). Fungitoxicity of 1,4-naphthoquinones to *Candida albicans* and *Trichophyton mentagrophytes*. *Can. J. Microbiol.* 21 1317–1321. 10.1139/m75-198 241479

[B10] Ghannoum M. Isham N. Verma A. Plaum S. Fleischer A. Hardas B. (2013). In vitro antifungal activity of naftifine hydrochloride against dermatophytes. *Antimicrob. Agents Chemother.* 57 4369–4372. 10.1128/aac.01084-13 23817365 PMC3754295

[B11] Ghannoum M. A. Rice L. B. (1999). Antifungal agents: mode of action, mechanisms of resistance, and correlation of these mechanisms with bacterial resistance. *Clin. Microbiol. Rev.* 12 501–517. 10.1128/cmr.12.4.501 10515900 PMC88922

[B12] Gow N. A. R. van de Veerdonk F. L. Brown A. J. P. Netea M. G. (2012). *Candida albicans* morphogenesis and host defense: discriminating invasion from colonization. *Nat. Rev. Microbiol.* 10 112–122. 10.1038/nrmicro2711 22158429 PMC3624162

[B13] Hassan S. T. S. Berchová-Bímová K. Petráš J. (2016). Plumbagin, a plant-derived compound, exhibits antifungal combinatory effect with amphotericin B against *Candida albicans* clinical isolates and anti-hepatitis C virus activity. *Phytother. Res.* 30 1487–1492. 10.1002/ptr.5650 27215409

[B14] Kano R. Hasegawa A. Watanabe S. Sato H. Nakamura Y. (2003). *Candida albicans* induced interleukin 8 production by human keratinocytes. *J. Dermatol. Sci.* 31 233–235. 10.1016/s0923-1811(03)00043-4 12727029

[B15] Kashem S. W. Kaplan D. H. (2016). Skin immunity to *Candida albicans*. *Trends Immunol.* 37 440–450. 10.1016/j.it.2016.04.007 27178391 PMC4931795

[B16] Kühbacher A. Burger-Kentischer A. Rupp S. (2017). Interaction of *Candida* species with the skin. *Microorganisms* 5:32. 10.3390/microorganisms5020032 28590443 PMC5488103

[B17] Larsen B. Petrovic M. De Seta F. (2018). Boric acid and commercial organoboron products as inhibitors of drug-resistant *Candida albicans*. *Mycopathologia* 183 349–357. 10.1007/s11046-017-0209-6 28993976

[B18] Lin Z. C. Hsieh P. W. Hwang T. L. Chen C. Y. Sung C. T. Fang J. Y. (2018). Topical application of anthranilate derivatives ameliorates psoriatic inflammation in a mouse model by inhibiting keratinocyte-derived chemokine expression and neutrophil infiltration. *FASEB J.* 32 6783–6795. 10.1096/fj.201800354 29920221

[B19] Lopez C. M. Wallich R. Riesbeck K. Skerka C. Zipfel P. F. (2014). *Candida albicans* uses the surface gpm1 to attach to human endothelial cells and to keratinocytes via the adhesive protein vitronectin. *PLoS One* 9:e90796 10.1371/journal.pone.090796 PMC3953207 24625558

[B20] Martinez-Rossi N. M. Peres N. T. Rossi A. (2017). Pathogenesis of dermatophytosis: sensing the host tissue. *Mycopathologia* 182 215–227. 10.1007/s11046-016-0057-9 27590362

[B21] Mathé L. Van Dijck P. (2013). Recent insights into *Candida albicans* biofilm resistance mechsnisms. *Curr. Genet.* 59 251–264. 10.1007/s00294-013-0400-3 23974350 PMC3824241

[B22] Meletiadis J. Chanock S. Walsh T. J. (2008). Defining targets for investigating the pharmacogenomics of adverse drug reactions to antifungal agents. *Pharmacogenomics* 9 561–584. 10.2217/14622416.9.5.561 18466103

[B23] Monika S. Małgorzata B. Zbigniew O. (2017). Contribution of aspartic proteases in *Candida* virulence. Protease inhibitors against *Candida* infections. *Curr. Protein Pept. Sci.* 18 1050–1062. 27514853 10.2174/1389203717666160809155749

[B24] Munawara U. Small A. G. Quach A. Gorgani N. N. Abbott C. A. Ferrante A. (2017). Cytokines regulate complement receptor immunoglobulin expression and phagocytosis of *Candida albicans* in human macrophages: a control point in anti-microbial immunity. *Sci. Rep.* 7:4050. 10.1038/s41598-017-04325-0 PMC5481325 28642550

[B25] Murali S. Aparna V. Suresh M. K. Biswas R. Jayakumar R. Sathianarayanan S. (2018). Amphotericin B loaded sulfonated chitosan nanoparticles for targeting macrophages to treat intracellular *Candida glabrata* infections. *Int. J. Biol. Macromol.* 110 133–139. 10.1016/j.ijbiomac.2018.01.028 29339278

[B26] Nagata K. Hirai K. I. Koyama J. Wada Y. Tamura T. (1998). Antimicrobial activity of novel furanonaphthoquinone analogs. *Antimicrib. Agents Chemother.* 42 700–702. 10.1128/aac.42.3.700 9517956 PMC105522

[B27] Neto J. B. A. da Silva C. R. Neta M. A. S. Campos R. S. Siebra J. T. Silva R. A. C. (2014). Antifungal activity of naphthoquinoidal compounds in vitro against fluconazole-resistant strains of different *Candida* species: a special emphasis on mechanisms of action on *Candida tropicalis*. *PLoS One* 9:e93698 10.1371/journal.pone.093698 PMC4015898 24817320

[B28] Ng S. M. S. Teo S. W. Yong Y. E. Ng F. M. Lau Q. Y. Jureen R. (2017). Preliminary investigations into developing all-*D* omiganan for treating mupirocin-resistant MRSA skin infections. *Chem. Biol. Drug Res.* 90 1155–1160. 10.1111/cbdd.13035 28581672

[B29] Park Y. N. Conway K. Pujol C. Daniels K. J. Soll D. R. (2020). EFG1 mutations, phenotypic switching, and colonization by clinical a/α strains of *Candida albicans*. *mSphere* 5:e0795-19. 10.1128/mSphere.00795-19 PMC7002308 32024711

[B30] Reis de Sá L. F. Toledo F. T. Gonçalves A. C. Sousa B. A. dos Santos A. A. Brasil P. F. (2017). Synthetic organotellurium compounds sensitize drug-resistant *Candida albicans* clinical isolates to fluconazole. *Antimicrob. Agents Chemother.* 61:e001231-16. 10.1128/AAC.01231-16 PMC5192104 27821447

[B31] Rejiniemon T. S. Arasu M. V. Duraipandiyan V. Ponmurugan K. Al-Dhabi N. A. Arokiyaraj S. (2014). In-vitro antimicrobial, antibiofilm, cytotoxic, antifeedant and larvicidal properties of novel quinine isolated from *Aegle marmelos* (Linn.) Correa. *Ann. Clin. Microbiol. Antimicrob.* 13:48. 10.1186/s12941-014-0048-y PMC4216832 25359605

[B32] Romo J. A. Pierce C. G. Chaturvedi A. K. Lazzell A. L. McHardy S. F. Saville S. P. (2017). Development of anti-virulence approaches for candidiasis via a novel series of small-molecule inhibitors of *Candida albicans* filamentation. *mBio* 8:e01991-17. 10.1128/mBio.01991-17 PMC5717394 29208749

[B33] Roscetto E. Contursi P. Vollaro A. Fusco S. Notomista E. Catania M. R. (2018). Antifungal and anti-biofilm activity of the first cryptic antimicrobial peptide from an archaeal protein against *Candida* spp. Clinical isolates. *Sci. Rep.* 8:17570. 10.1038/s41598-018-35530-0 PMC6279838 30514888

[B34] Shiraki Y. Ishibashi Y. Hiruma M. Nishikawa A. Ikeda S. (2008). *Candida albicans* abrogates the expression of interferon-γ-inducible protein-10 in human keratinocytes. *FEMS Immunol. Med. Microbiol.* 54 122–128. 10.1111/j.1574-695x.2008.00457.x 18647352

[B35] Tsai P. C. Chu C. L. Fu Y. S. Tseng C. H. Chen Y. L. Chang L. S. (2014). Naphtho[1,2-*b*]furan-4,5-dione inhibits MDA-MB-231 cell migration and invasion by suppressing Src-mediated signaling pathways. *Mol. Cell. Biochem.* 387 101–111. 10.1007/s11010-013-1875-4 24162594

[B36] Tsang N. Y. Chik W. I. Sze L. P. Wang M. Z. Tsang S. W. Zhang H. J. (2018). The use of naphthoquinones and furano-naphthoquinones as antiinvasive agents. *Curr. Med. Chem.* 25 5007–5056. 10.2174/0929867324666171006131927 28990521

[B37] Tseng C. H. Chen Y. L. Yang S. H. Peng S. I. Cheng C. M. Han C. H. (2010). Synthesis and antiproliferative evaluation of certain iminonaphtho[2,3-*b*]furan derivatives. *Bioorg. Med. Chem.* 18 5172–5182. 10.1016/j.bmc.2010.05.062 20591678

[B38] Tseng C. H. Lin C. S. Shih P. K. Tsao L. T. Wang J. P. Cheng C. M. (2009). Furo[3’,2’:3,4]naphtha[1,2-*d*]imidazole derivatives as potential inhibitors of inflammatory factors in sepsis. *Bioorg. Med. Chem.* 17 6773–6779. 10.1016/j.bmc.2009.07.054 19699097

[B39] Vavala E. Colone M. Passariello C. Celestino I. Toccacieli L. Stringaro A. (2013). Characterization of biofilms in drug-sensitive and drug-resistant strains of *Candida albicans*. *J. Chemother.* 25 87–95. 10.1179/1973947812y.0000000047 23684356

[B40] Wall G. Montelongo-Jauregui D. Bonifacio B. V. Lopez-Ribot J. L. Uppuluri P. (2019). *Candida albicans* biofilm growth and dispersal: contributions to pathogenesis. *Curr. Opin. Microbiol.* 52 1–6. 10.1016/j.mib.2019.04.001 31085405 PMC6842673

[B41] Wartenberg A. Linde J. Martin R. Schreiner M. Horn F. Jacobsen I. D. (2014). Microevolution of *Candida albicans* in macrophages restores filamentation in a nonfilamentous mutant. *PLoS Genet.* 10:e1004824 10.1371/journal.pone.1004824 PMC4256171 25474009

[B42] Xie F. Chang W. Zhang M. Li Y. Li W. Shi H. (2016). Quinone derivatives isolated from the endolichenic fungus *Phialocephala fortinii* are Mdr1 modulators that combat azole resistance in *Candida albicans*. *Sci. Rep.* 6:33687. 10.1038/srep33687 PMC5030645 27650180

[B43] Yang S. C. Aljuffali I. A. Sung C. T. Lin C. F. Fang J. Y. (2016). Antimicrobial activity of topically-applied soyaethyl morpholinium ethosulfate micelles against *Staphylococcus* species. *Nanomedicine* 11 657–671. 10.2217/nnm.15.217 26911580

[B44] Yang S. C. Tang K. W. Lin C. H. Alalaiwe A. Tseng C. H. Fang J. Y. (2019). Discovery of furanoquinone derivatives as a novel class of DNA polymerase and gyrase inhibitors for MRSA eradication in cutaneous infection. *Front. Microbiol.* 10:1197 10.3389/fmicb.2018.1197 PMC6549599 31191504

[B45] Yang S. C. Yen F. L. Wang P. W. Aljuffali I. A. Weng Y. H. Tseng C. H. (2017). Naphtho[1,2-*b*]furan-4,5-dione is a potent anti-MRSA agent against planktonic, biofilm, and intracellular bacteria. *Future Microbiol.* 12 1059–1073. 10.2217/fmb-2017-0044 28799789

[B46] Zeidler U. Lettner T. Lassnig C. Muller M. Lajko R. Hintner H. (2009). UME6 is a crucial downstream target of other transcriptional regulators of true hyphal development in *Candida albicans*. *FEMS Yeast Res.* 9 126–142. 10.1111/j.1567-1364.2008.00459.x 19054126

[B47] Zhang X. Li T. Chen X. Wang S. Liu Z. (2018). Nystatin enhances the immune response against *Candida albicans* and protects the ultrastucture of the vaginal epithelium in a rat model of vulvovaginal candidiasis. *BMC Microbiol.* 18:166. 10.1186/s12866-018-1316-3 30359236 PMC6202846

