# Supplementary figures and images for: Synthetic Naphthofuranquinone Derivatives Are Effective in Eliminating Drug-Resistant Candida albicans in Hyphal, Biofilm, and Intracellular Forms: An Application for Skin-Infection Treatment
Source: Front Microbiol. 2020 Aug 26;11:2053. doi: 10.3389/fmicb.2020.02053 (PMC7479094; doi:10.3389/fmicb.2020.02053)

Suppl. Fig. 1

(A)

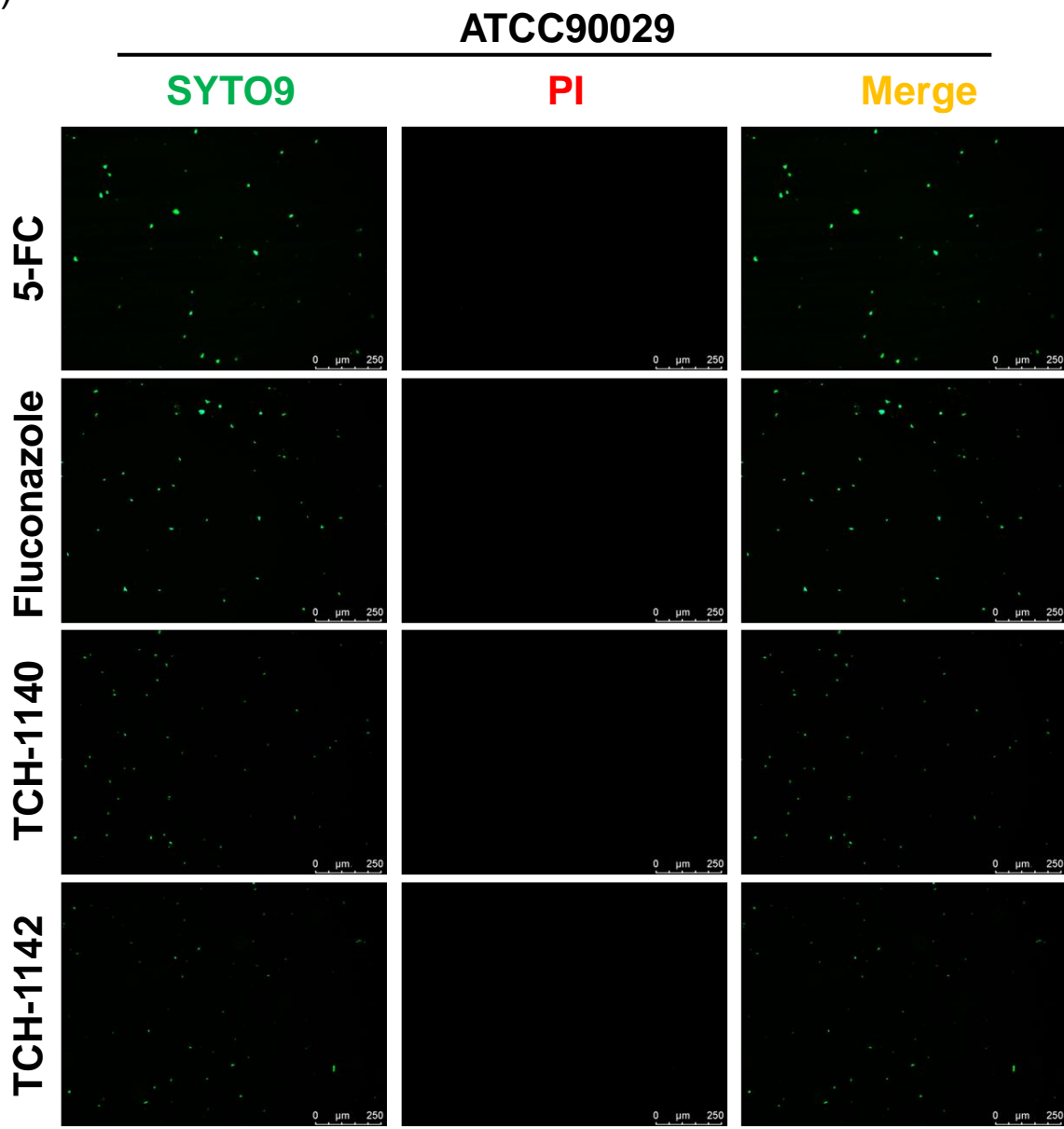

(B)

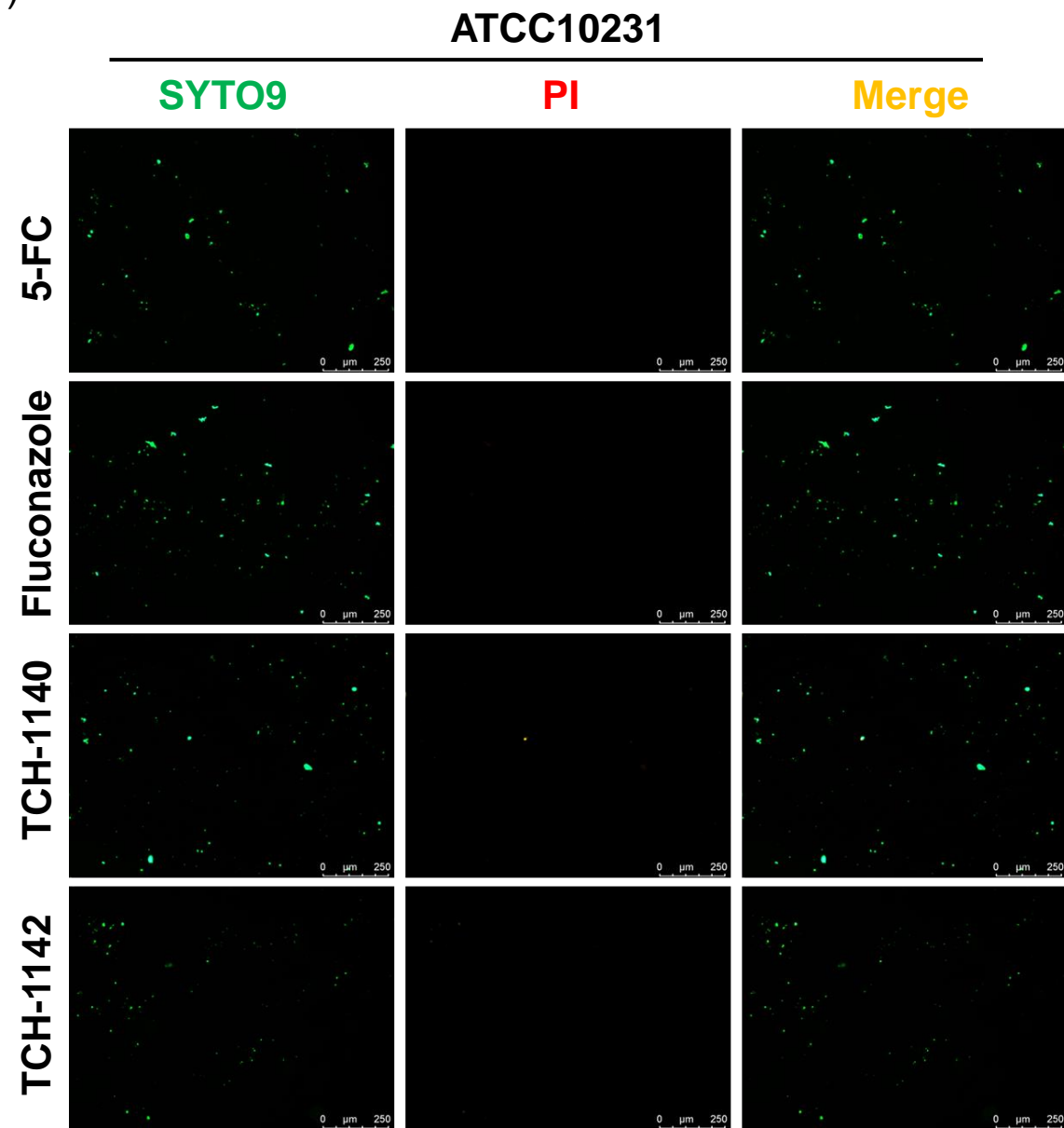

(C)

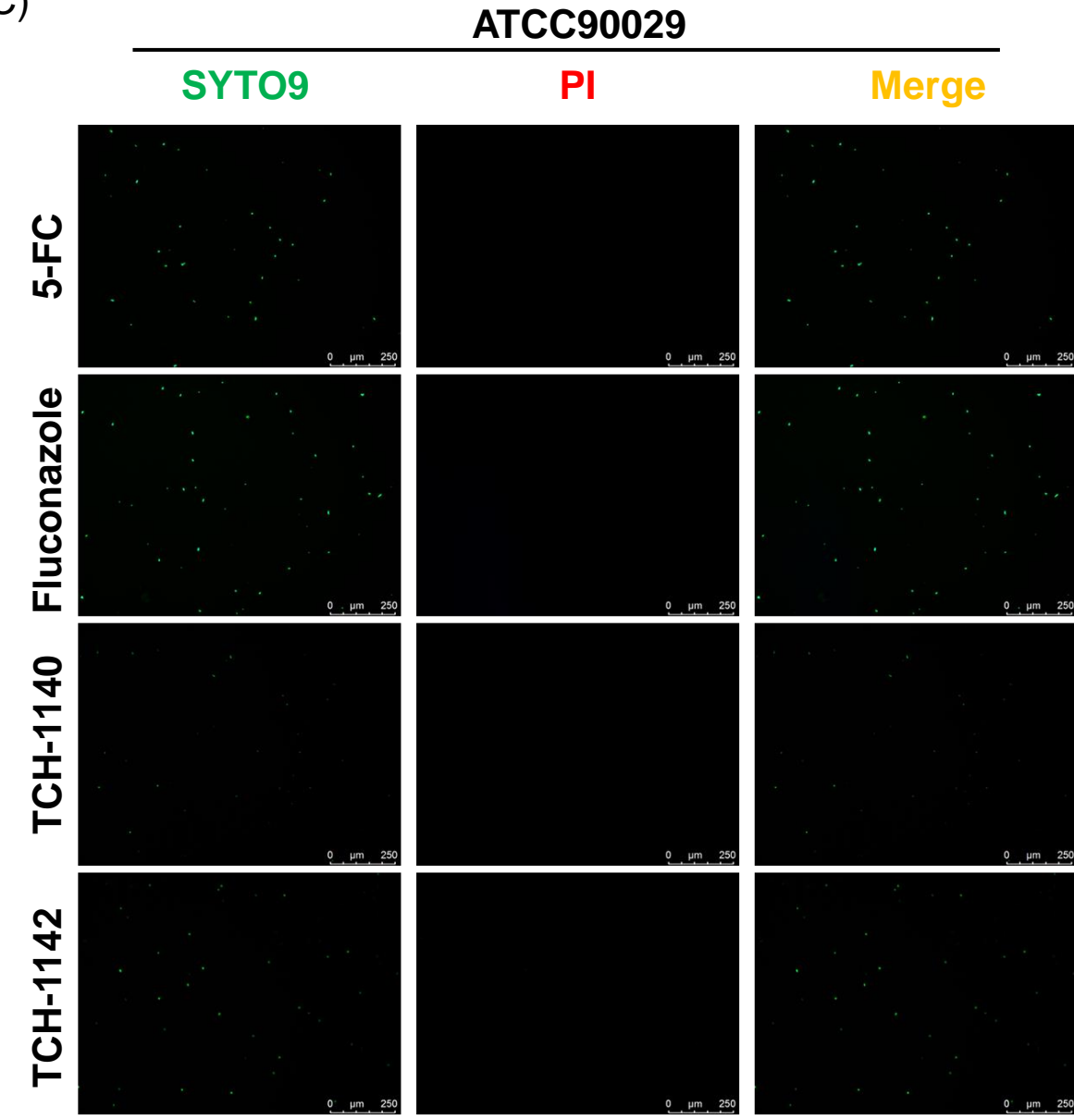

(D)

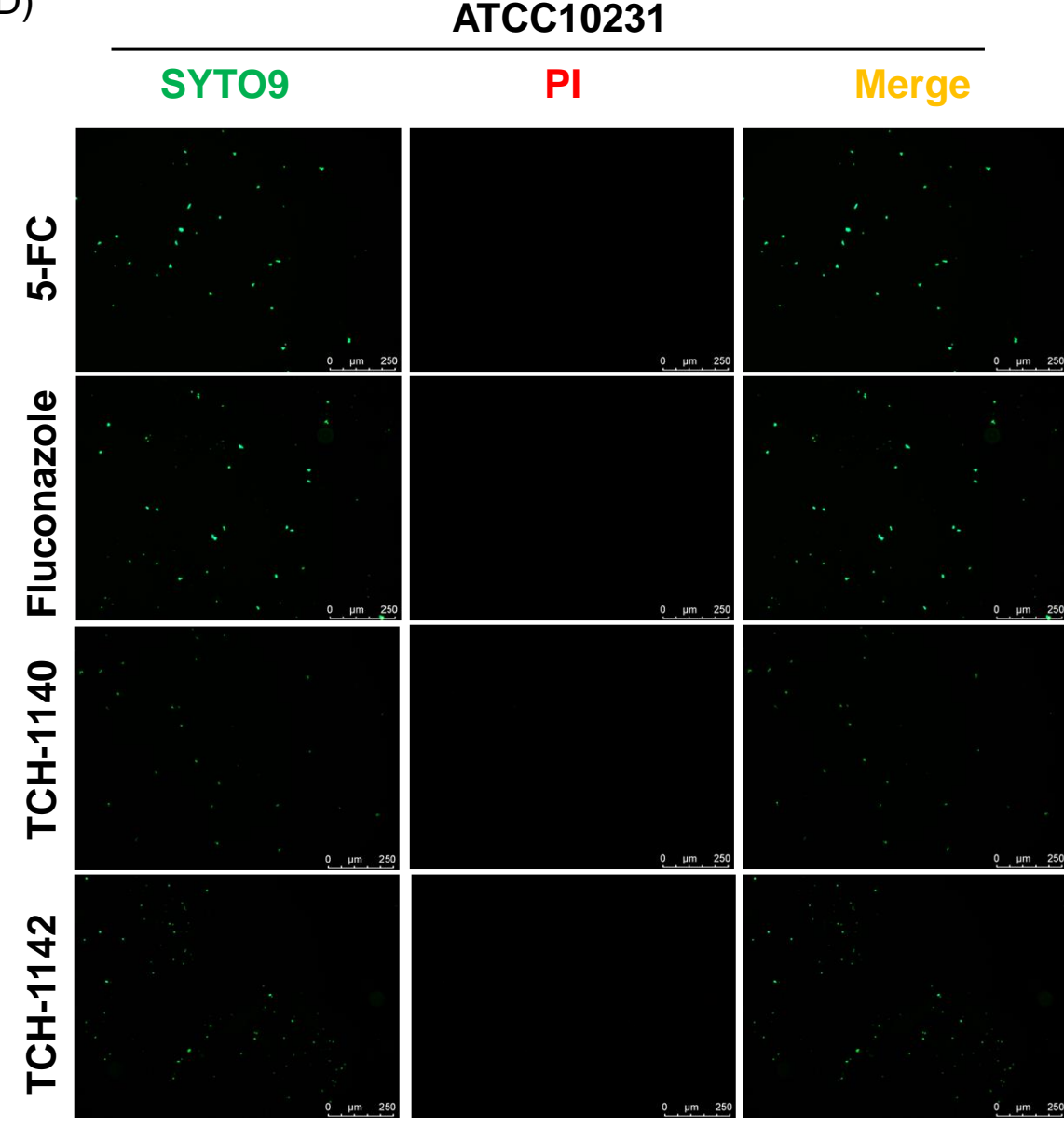

Supplement: FIGURE S1 — The planktonic live/dead C. albicans strains treated by the agents at 46.9 or 93.8 μM viewed under fluorescence microscopy: (A) ATCC90029 treated by 5-FC, fluconazole, and naphthofuranquinones at 46.9 μM. (B) ATCC10231 treated by 5-FC, fluconazole, and naphthofuranquinones at 46.9 μM. (C) ATCC90029 treated by 5-FC, fluconazole, and naphthofuranquinones at 93.8 μM. (D) ATCC10231 treated by 5-FC, fluconazole, and naphthofuranquinones at 93.8 μM. [file Image_1.pdf]

Suppl. Fig. 2

(A)

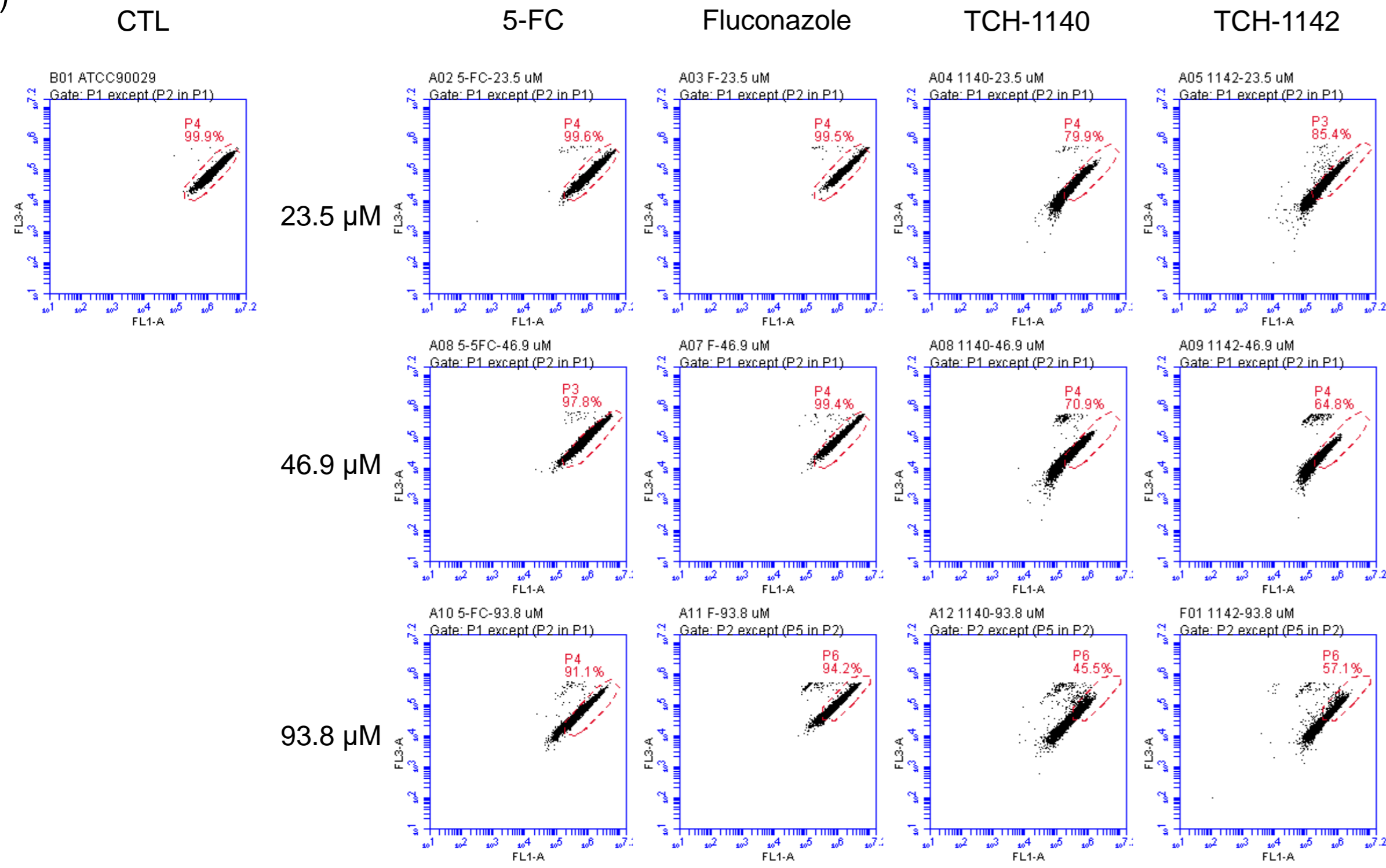

(B)

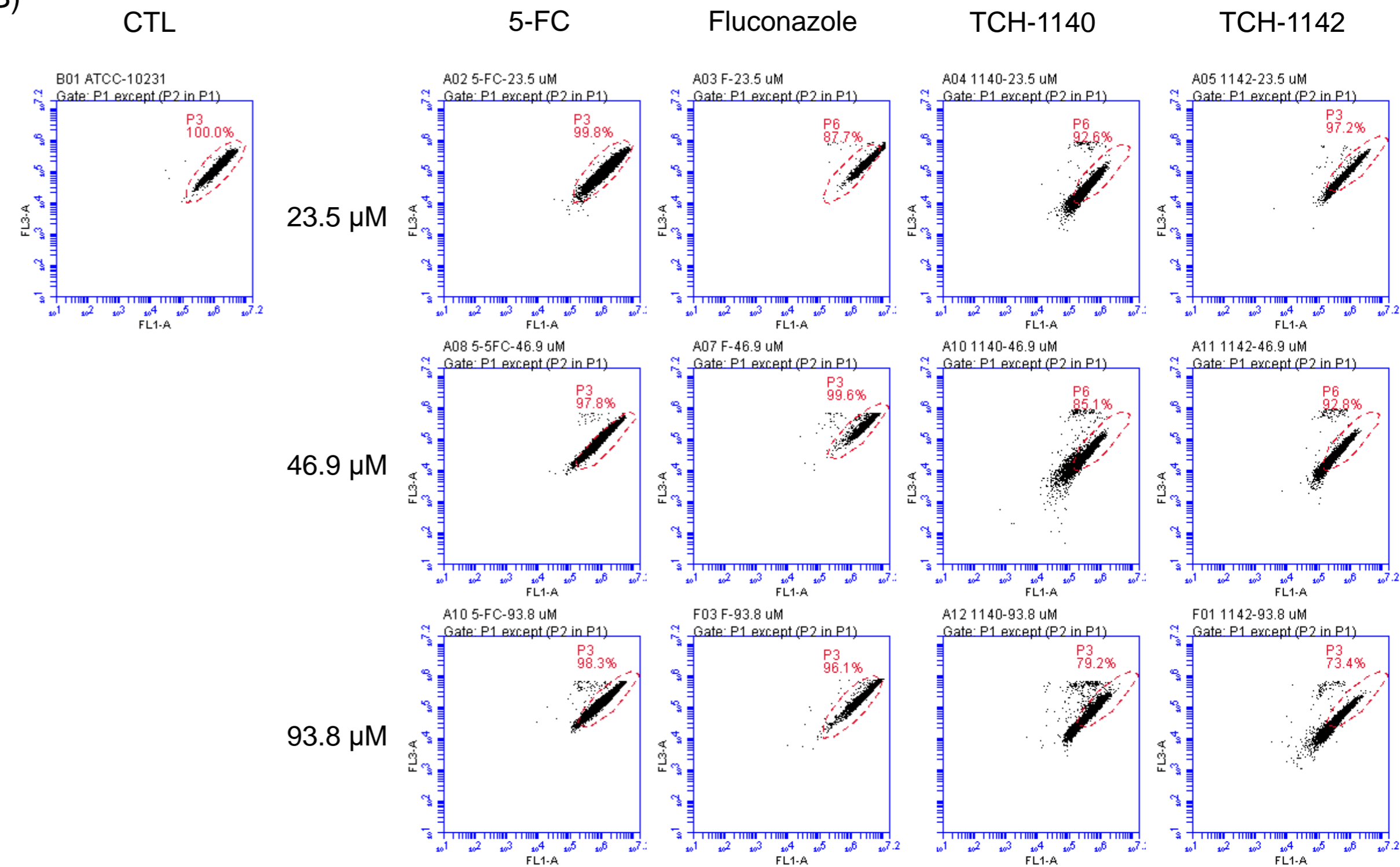

Supplement: FIGURE S2 — The flow cytometry of C. albicans viability treated by 5-FC, fluconazole, and naphthofuranquinones : (A) ATCC90029; and (B) ATCC10231. FL1 (x-axis) represents SYTO9 staining. FL3 (y-axis) represents PI staining. [file Image_2.pdf]

Suppl. Fig. 3

(A)

HaCaT

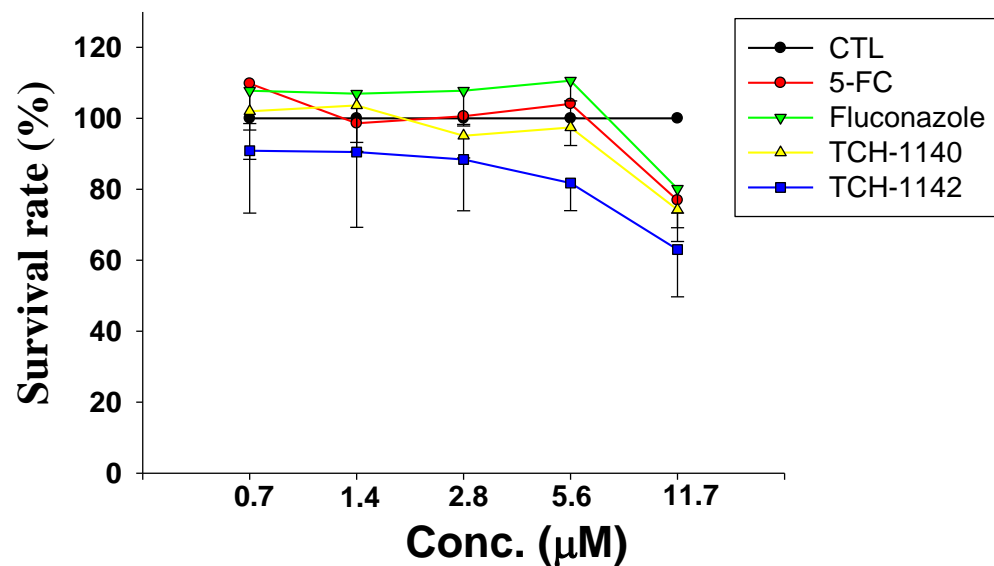

(B)

THP-1

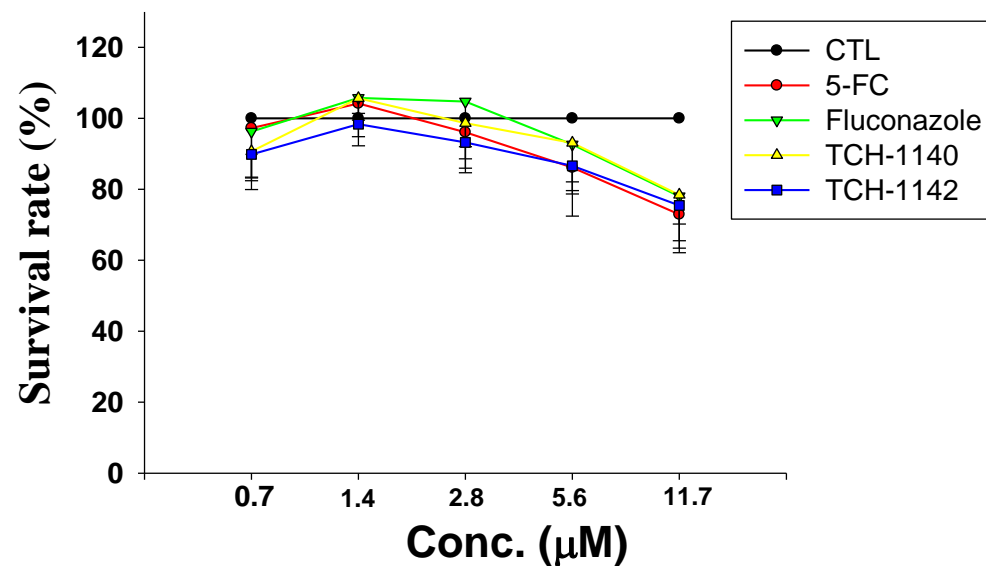

Supplement: FIGURE S3 — The survival rate of keratinocytes and macrophages treated by 5-FC, fluconazole, and naphthofuranquinones measured by CCK-8 assay: (A) keratinocytes; and (B) macrophages. All data are presented as the mean of three experiments ±S.D. [file Image_3.pdf]

Suppl. Fig. 5

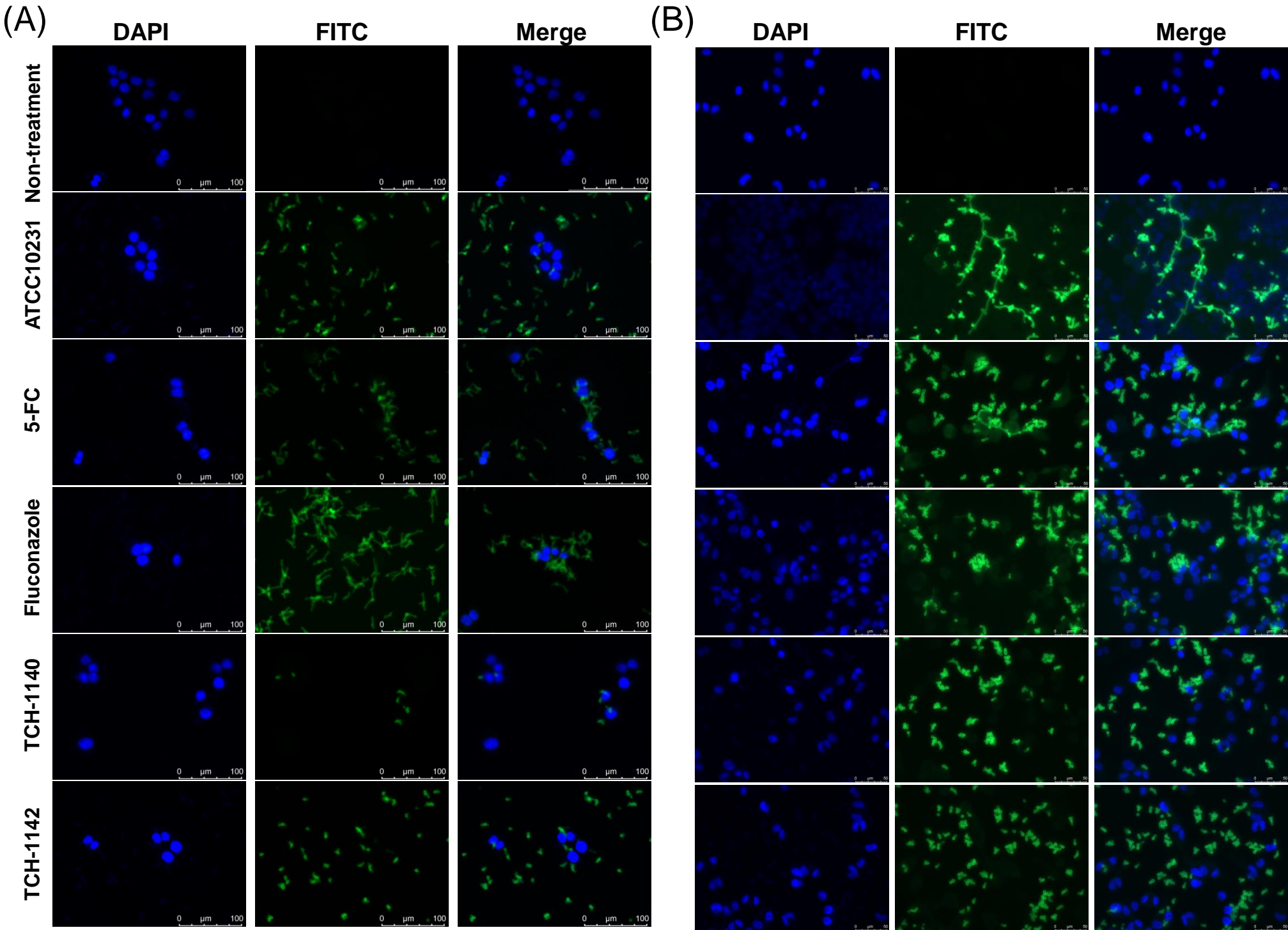

Supplement: FIGURE S5 — The large field view of confocal microscopic images of ATCC10231 after treatment of 5-FC, fluconazole, and naphthofuranquinones: (A) ATCC10231-infected keratinocytes; and (B) ATCC10231-infected macrophages. [file Image_5.pdf]

Suppl. Fig. 6

(A)

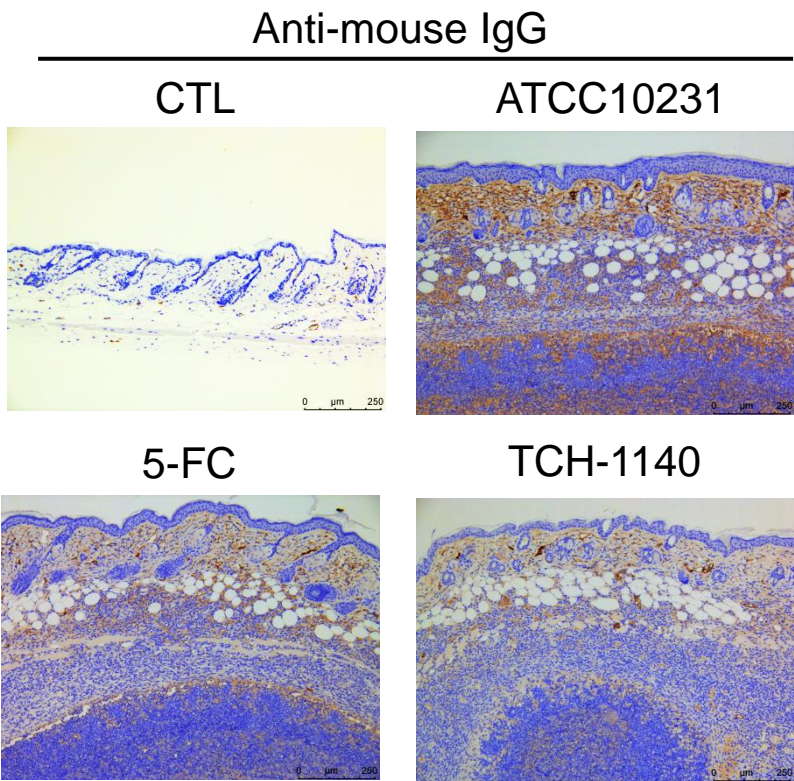

(B)

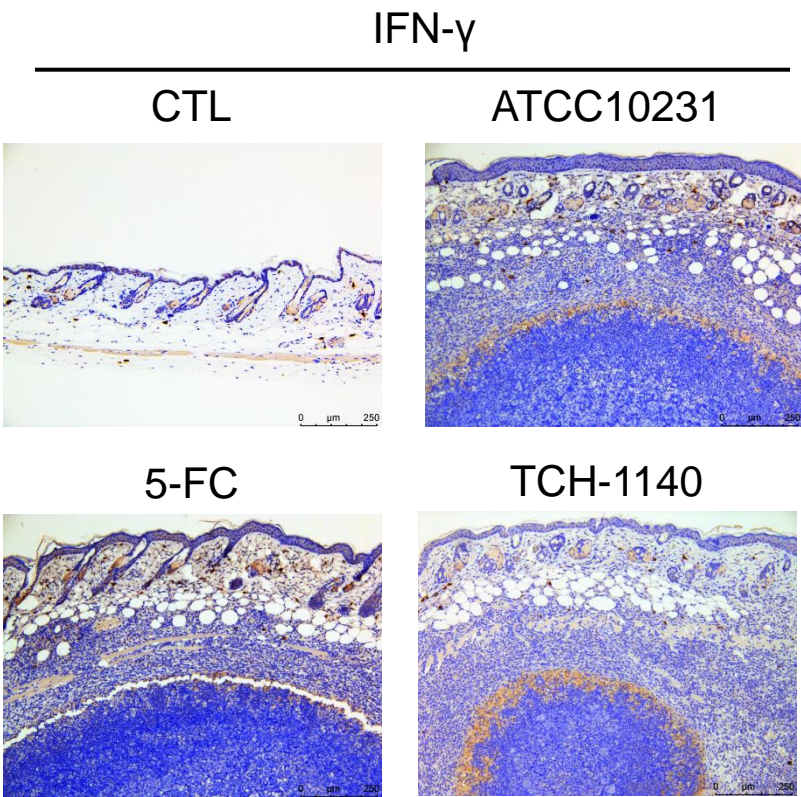

(C)

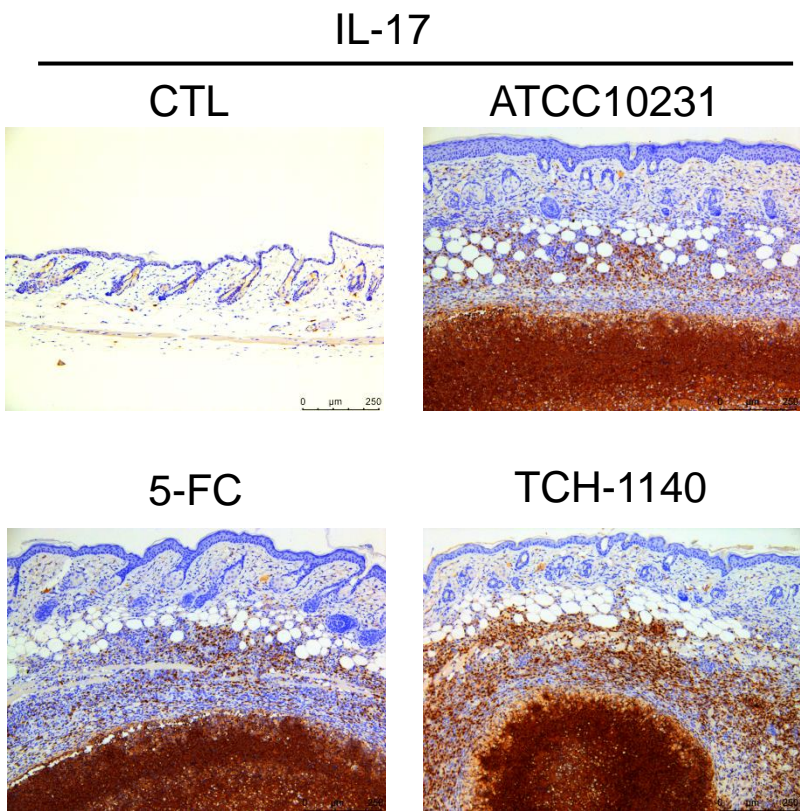

(D)

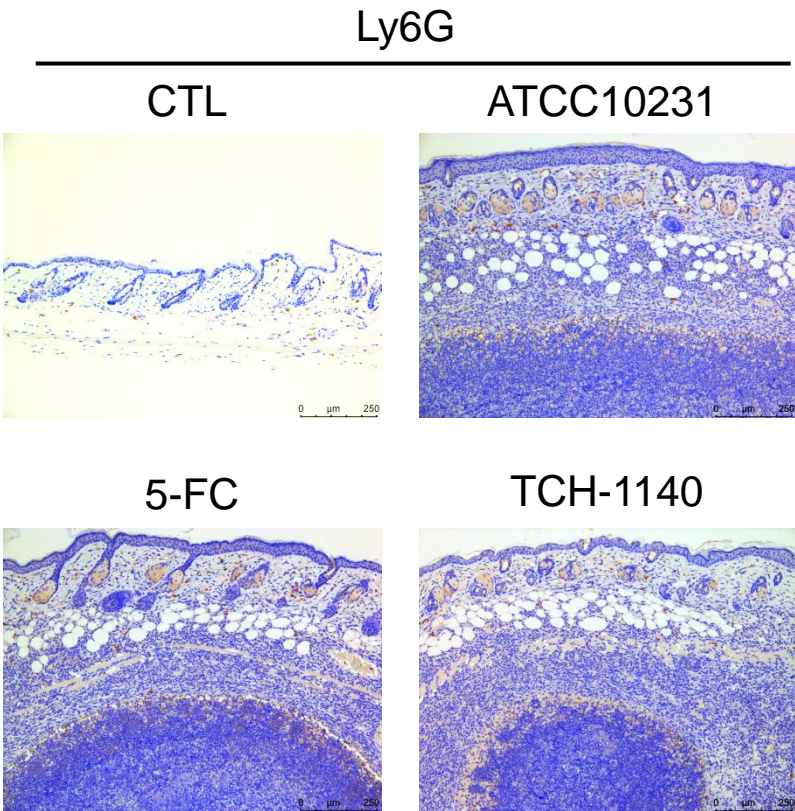

Supplement: FIGURE S6 — The IHC staining of ATCC10231-infected mouse skin after treatment of 5-FC or TCH-1140: (A) IgG; (B) IFN-γ; (C) IL-17, and (D) Ly6G. [file Image_6.pdf]
