# Supplementary material for: Synthetic Naphthofuranquinone Derivatives Are Effective in Eliminating Drug-Resistant Candida albicans in Hyphal, Biofilm, and Intracellular Forms: An Application for Skin-Infection Treatment
Source: Front Microbiol. 2020 Aug 26;11:2053. doi: 10.3389/fmicb.2020.02053 (PMC7479094; doi:10.3389/fmicb.2020.02053)
Supplement: FIGURE S4 — The flow cytometry of keratinocytes and C. albicans stained by Annexin V and PI to detect the early or late apoptosis: (A) keratinocytes treated with TCH-1140 at 93.8 μM; (B) keratinocytes treated with TCH-1142 at 93.8 μM; (C) ATCC90029 treated with TCH-1140 at 23.5 μM; (D) ATCC10231 treated with TCH-1140 at 23.5 μM; (E) ATCC90029 treated with TCH-1142 at 23.5 μM; and (F) ATCC10231 treated with TCH-1142 at 23.5 μM. All data are presented as the mean of three experiments ±S.D. LL, lower left; LR, lower right; UL, upper left; UR, upper right. [file Image_4.pdf]

Suppl. Fig. 4

(A) **HaCaT** **HaCaT/TCH1140**

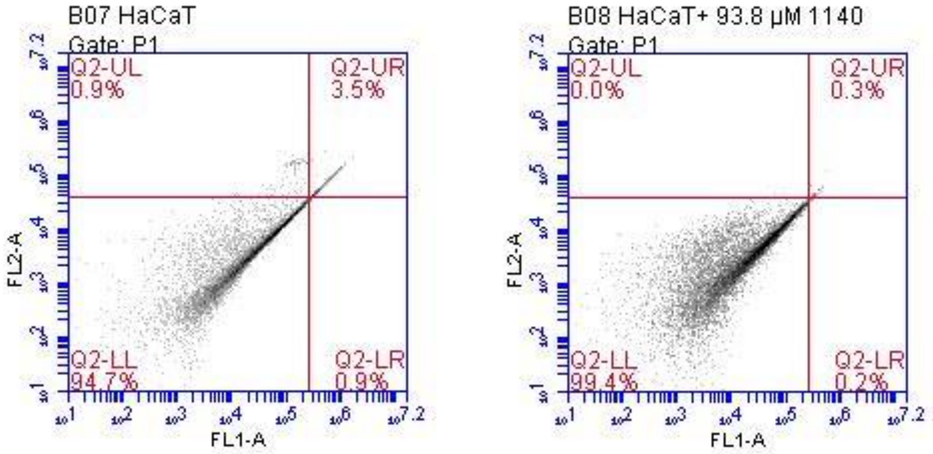

	LL	LR	UL	UR	UL+UR+LR
CTL	94.8±0.1	0.8±0.2	0.6±0.3	3.8±0.3	5.2±0.1
TCH1140	99.5±0.1	0.2±0.0	0.1 ±0.1	0.2±0.1	0.5±0.1

(B) **HaCaT** **HaCaT/TCH1142**

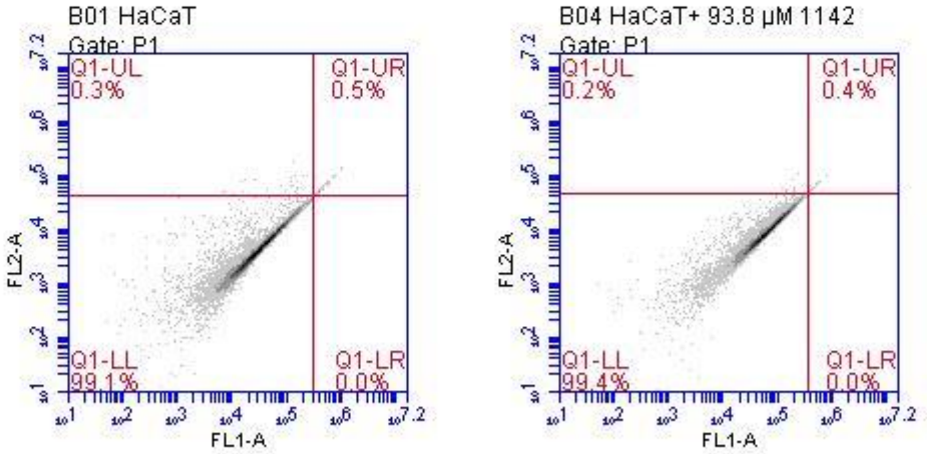

	LL	LR	UL	UR	UL+UR+LR
CTL	98.4±0.8	0.5±0.8	0.4±0.2	0.7±0.3	1.6±0.8
TCH1142	99.5±0.3	0.1±0.1	0.2±0.2	0.2±0.2	0.4±0.4

(C) **ATCC90029** **ATCC90029/TCH1140**

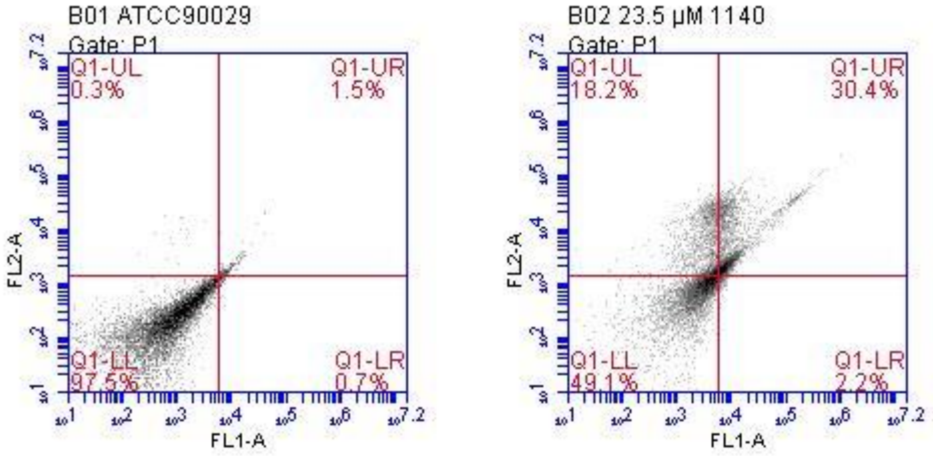

	LL	LR	UL	UR	UL+UR+LR
CTL	98.0±0.9	0.4±0.4	0.6±0.4	1.0± 0.8	2.0±0.8
TCH1140	47.3±18.7	2.3±0.8	16.4±8.9	34.0±26.8	52.7±18.7

(D) **ATCC10231** **ATCC10231/TCH1140**

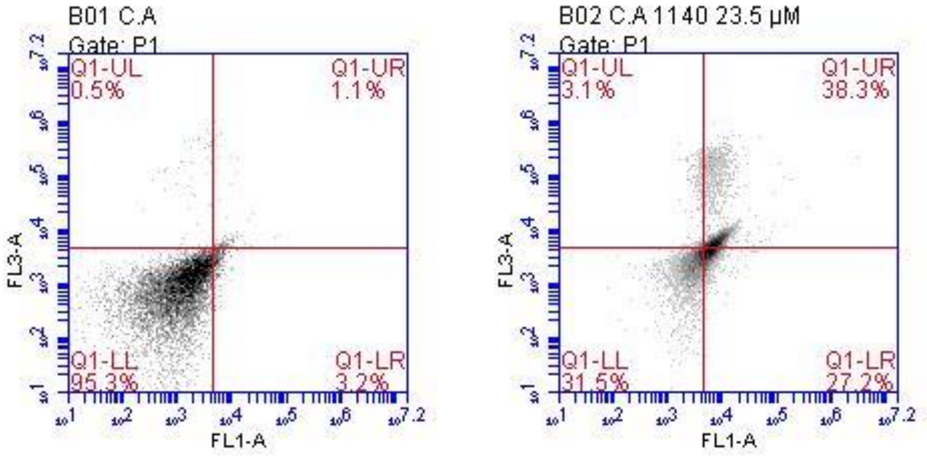

	LL	LR	UL	UR	UL+UR+LR
CTL	98.9±0.4	0.4±0.7	0.7±0.4	0.6±0.5	1.8±1.5
TCH1140	54.5±19.5	4.5±3.8	9.1±5.1	31.9±20.4	45.4±19.4

(E) **ATCC90029** **ATCC90029/TCH1142**

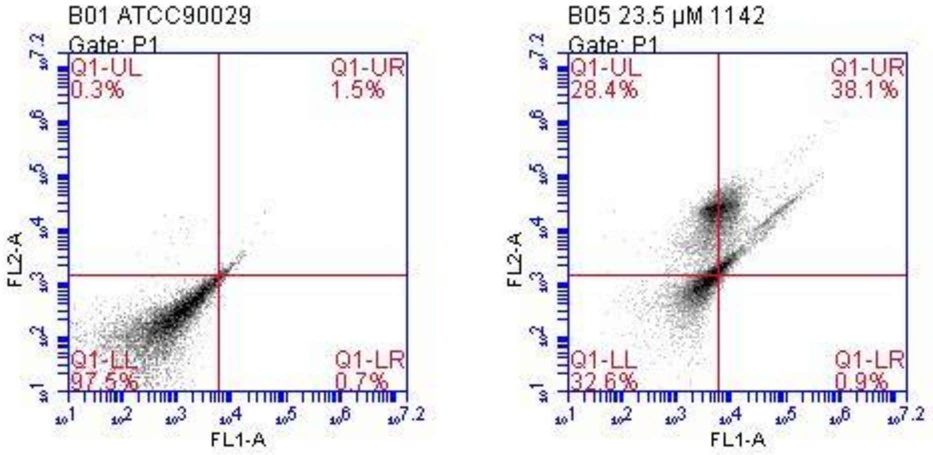

	LL	LR	UL	UR	UL+UR+LR
CTL	98.0±0.9	0.4±0.4	0.6±0.4	1.0±0.8	2.0±0.8
TCH1142	45.3±23.1	2.3±1.8	19.2±8.6	33.2±22.7	54.7±23.0

(F) **ATCC10231** **ATCC10231/TCH1142**

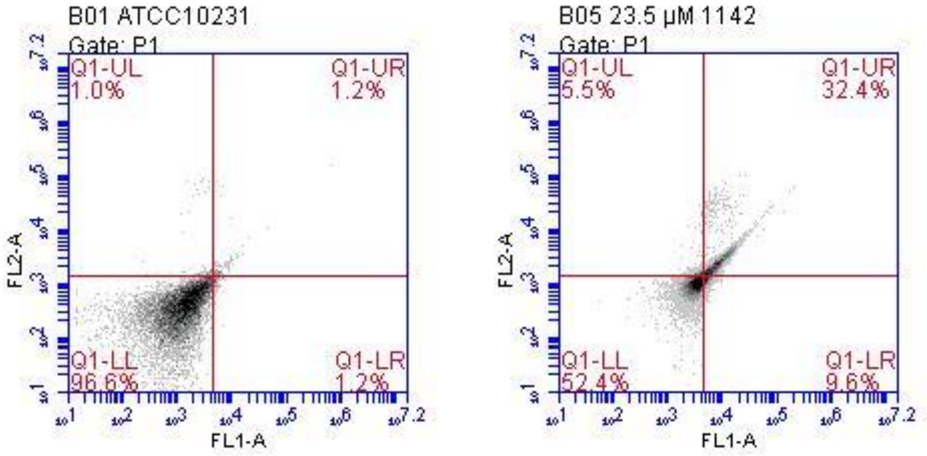

	LL	LR	UL	UR	UL+UR+LR
CTL	98.9±0.4	0.4±0.7	0.7±0.4	0.6±0.5	1.8±1.5
TCH1142	61.6±7.9	4.7±4.4	8.7±2.9	25.0±6.7	38.4±7.9
