# Supplementary material for: Synthetic Naphthofuranquinone Derivatives Are Effective in Eliminating Drug-Resistant Candida albicans in Hyphal, Biofilm, and Intracellular Forms: An Application for Skin-Infection Treatment
Source: Front Microbiol. 2020 Aug 26;11:2053. doi: 10.3389/fmicb.2020.02053 (PMC7479094; doi:10.3389/fmicb.2020.02053)
Supplement: Supplementary file 7 [file Table_1.pdf]

Suppl. Table 1. The MFC of naphthofuranquinones against ATCC10231

Compounds	Structure	MFC (μM)
TCH-1139	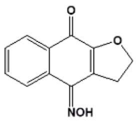	1465.8
TCH-1140	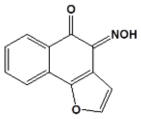	2.9
TCH-1142	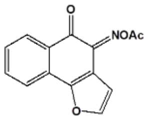	2.4
TCH-1181	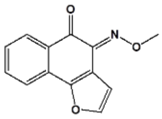	5501.3
TCH-1182	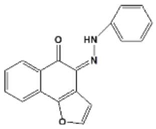	2167.9
TCH-1187	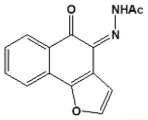	2458.3
TCH-1190	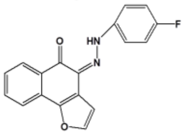	2040.6
TCH-1198	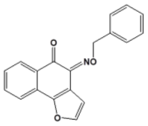	4121.2
TCH-1199	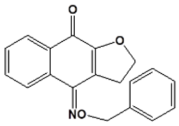	1030.3
TCH-2958	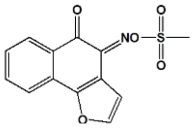	268.2
TCH-5261	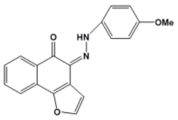	245.4
TCH-5262	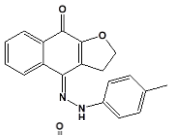	2067.3
TCH-5263	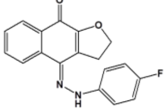	2040.6
TCH-5264	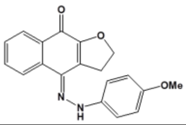	1963.4

MFC, minimum fungicidal concentration.

Each value represents the 3 replicates.
